# Is Diet Flexibility an Adaptive Life Trait for Relictual and Peri-Urban Populations of the Endangered Primate *Macaca sylvanus*?

**DOI:** 10.1371/journal.pone.0118596

**Published:** 2015-02-25

**Authors:** Yasmina Maibeche, Aissa Moali, Nassima Yahi, Nelly Menard

**Affiliations:** 1 Laboratory of Ecology and Environment, Faculty of Natural Sciences and Life, University Abderahmane Mira, Bejaia, Algeria; 2 Laboratory of vegetal ecology, Faculty of Biological Sciences, University of Sciences and Technology Houari Boumediene, Alger, Algeria; 3 UMR 6553, laboratoire “Ecosystems, Biodiversity, Evolution”, Centre National de la Recherche Scientifique/University of Rennes 1, Station Biologique de Paimpont, Paimpont, France; Institut Pluridisciplinaire Hubert Curien, FRANCE

## Abstract

Habitat loss, fragmentation and urban expansion may drive some species to marginal habitats while others succeed in exploiting urban areas. Species that show dietary flexibility are more able to take advantage of human activities to supplement their diet with anthropogenically abundant and accessible resources. The Barbary macaque (*Macaca sylvanus*) is an endangered species due to the loss of its habitat, and human pressure. The population of Gouraya National Park (Algeria) lives in a relictual habitat that constitutes about 0.6% of the species range. In addition, this population is a unique case where urban expansion favours contact zones between Barbary macaque habitats and a big city (Bejaia). We quantified the dietary composition of Gouraya macaques over an annual cycle with the objective to understand how diet flexibility of this species may help it adapt to a relictual habitat or cope with urban expansion. We recorded the phenology of plant species every month. This study shows that Gouraya macaques, compared to those living in other forest types of the distribution area, are under lower seasonal constraints. They consume a greater amount of fruit and seeds that are available throughout much of the year, and a lesser amount of costly to find and extract subterranean foods. Therefore the Gouraya relictual habitat appears as a favourable environment compared to other major habitats of that species. This study also shows that colonizing peri-urban zones increases the availability and species richness of diet resources for Barbary macaques as they consume more human foods and exotic plants than in farther sites. Adult males eat more human foods than adult females and immatures do. The exploitation of high-energy anthropogenic food could favour macaque population growth and expansion towards the city center associated with human/macaque conflicts. We recommend applying management actions to restore macaques back to their natural habitat.

## Introduction

The global expansion of human populations is associated with more intense land use (agriculture, presence of domestic livestock) and zones of more frequent contact between urbanites and wildlife. The consequences for wildlife in terms of reduced quality, loss and fragmentation of habitat are well-documented [1]. One of the main consequences of habitat alteration is decreased availability of resources. To deal with such changes, wild animals may show flexibility in how they exploit resources (*e*.*g*. diets, home ranges) [2,3]. Species that are specialists, regarding diet and/or habitat use, are more vulnerable to habitat modifications than generalists because they are less able to adapt [4,5]. For instance, forest-specialists, which avoid exploiting matrix habitats and are relatively unable to disperse among forest patches, may be particularly vulnerable [6].

The development of urban areas is expected to affect biodiversity and to cause more frequent human-wildlife conflicts [7,8]. Some species avoid urban areas and will not persist in such altered environments [7,9], whereas others can occasionally exploit cities or permanently live in them [10–12]. Occasional exploiters can adapt to urban life, and they seize the opportunity to exploit new resources and use anthropogenic food [8,13].

Studies of the ecology of wild animals at the edge of the species’ range or in marginal or anthropogenically-altered habitats provide opportunities to estimate their resilience to habitat disturbance. In the case of endangered species, dietary breadth data may be critical to understanding their ability to adapt to a novel or rapidly changing environment and to help in political management and conservation planning.

Primates are heavily affected by human activities, with almost half of all species classified as endangered or critically endangered due to the disappearance or the degradation of their habitats [14,15]. How primates respond to habitat modification may depend on the ecological behaviour of each species. Consequently, primatologists are increasingly interested in monkeys’ responses to anthropogenic habitat alteration [3,16,17], and some studies document monkeys’ ecological behaviours in a variety of habitats within their area of distribution [18–20].

Many primate species include populations that may take advantage of the proximity to humans (*e*.*g*. crop raiding) to supplement their diets with abundant and accessible food items that contain more digestible carbohydrates and less fibre and toxins compared to foods found in the wild [21–23]. In particular, primates living in urban areas may eat garden plants and/or be deliberately fed by city dwellers or by tourists [24,25]. As human population growth and urban expansion lead to increasing opportunities for encounters (and potential conflicts) between monkeys and humans, it has become critical to study monkeys in cities [26]. Despite a growing interest, ecological studies of urban monkeys remain scarce. They primarily focus on Asian macaques (*Macaca fascicularis*, [27,28], *M*. *radiata*, [29,30]), chacma baboons (*Papio ursinus*, [31]) and purple-faced langurs (*Semnopithecus vetulus nestor*, [25,32–34]), and to a lesser extent on New World primates (capuchins *Cebus libidinosus*, [35], marmosets *Callithrixpenicillata*, [36]).

Among the 19 species of macaques, Richard [37] distinguished between two ecological groups (weed and non-weed macaques) according to their ability to compete with people through much of their range (weeds) or their reliance upon forests where they have little or no contact with people (non-weeds). Accordingly, social groups of weed species, such as *M*. *fascicularis*, *M*. *radiata*, *M*. *sinica*, and *M*. *mulatta* are able to live in urban areas, whereas non-weed species, which include the Barbary macaque (*M*. *sylvanus*, [37]) are not.

As a non-weed species, the Barbary macaque should be highly sensitive to human expansion (and associated forest loss and degradation and urban expansion). Investigating the resilience of this species to habitat changes requires assessing its ecological flexibility in the different contexts of marginal habitats, human proximity and/or urban areas. The Barbary macaque is listed as “Endangered” on the Red List of Threatened Species, Appendix II of CITES [15]. Almost all wild Barbary macaques live in mountainous areas, specifically, in temperate mixed cedar-oak forests of Algeria and Morocco [38]. Their diets in most major forest types of their home-ranges have been well-studied [39–41]. These macaques exhibit a generalist feeding behaviour. They consume various parts of a wide range of plant species. Their diets are highly flexible according to variations in resource availability induced by the high seasonality in temperate habitats [42] or by human habitat exploitation [39]. In contrast to their dietary flexibility, Barbary macaques are forest-dependent and avoid crossing open areas [43]. That is probably why, unlike other macaque species that occasionally or permanently exploit cities [24,28,37], wild Barbary macaques have stayed away from cities so far, except for artificially maintained macaques in Gibraltar [44].

The main objectives of this study are to better understand how the ecological flexibility of the Barbary macaque may help it live in marginal habitats, and potentially cope with urban expansion. We worked in Gouraya National Park, where this species lives in a relictual Mediterranean thermophilous scrub habitat at the eastern edge of its range, near sea level, and in the vicinity of a large and expanding city (Bejaia). This population is presently isolated from other populations as a result of the disappearance of forest areas and the expansion of cultivated areas. Firstly, our study is a first examination of Barbary macaque diets and of resource availability in this relictual habitat. It aims at assessing the ecological flexibility of the species by comparing macaques diets in Gouraya with those of conspecifics in other habitats. Secondly, we hypothesized that Barbary macaques grew adapted to urban areas by modifying their modalities of resource exploitation, especially their diets. In the Gouraya population, almost all social groups have various degrees of contact with humans in residential areas or areas frequented by tourists. To our knowledge, this is the only context in which the home ranges of some groups of wild Barbary macaques overlap peri-urban areas. We predicted that distance from urban areas affects dietary composition, with a greater proportion of exotic vegetation and human foods in the vicinity of the city. Assuming that getting food from humans is risky and/or linked to inter-individual dominance hierarchy, we expected that dietary composition would depend on the classes of individuals. Specifically, we predicted that adult males are more able to exploit human food because they are (1) less vulnerable to human harassment and/or (2) dominant over females and immatures in their access to preferred resources, as shown in provisioned populations in Gibraltar [45].

## Materials and Methods

### Ethics statement

This research complies with the ethics guidelines of the CNRS/ University of Rennes, the University of Bejaia and the University of Algiers. This was an observational study. Observers remained quiet during observations and never tried to be in close contact with the macaques. Permission for this study was obtained from the National Park of Gouraya and the Ministry of Agriculture of Algeria.

### Study sites, macaque groups and periods of observation

**Fig 1:**
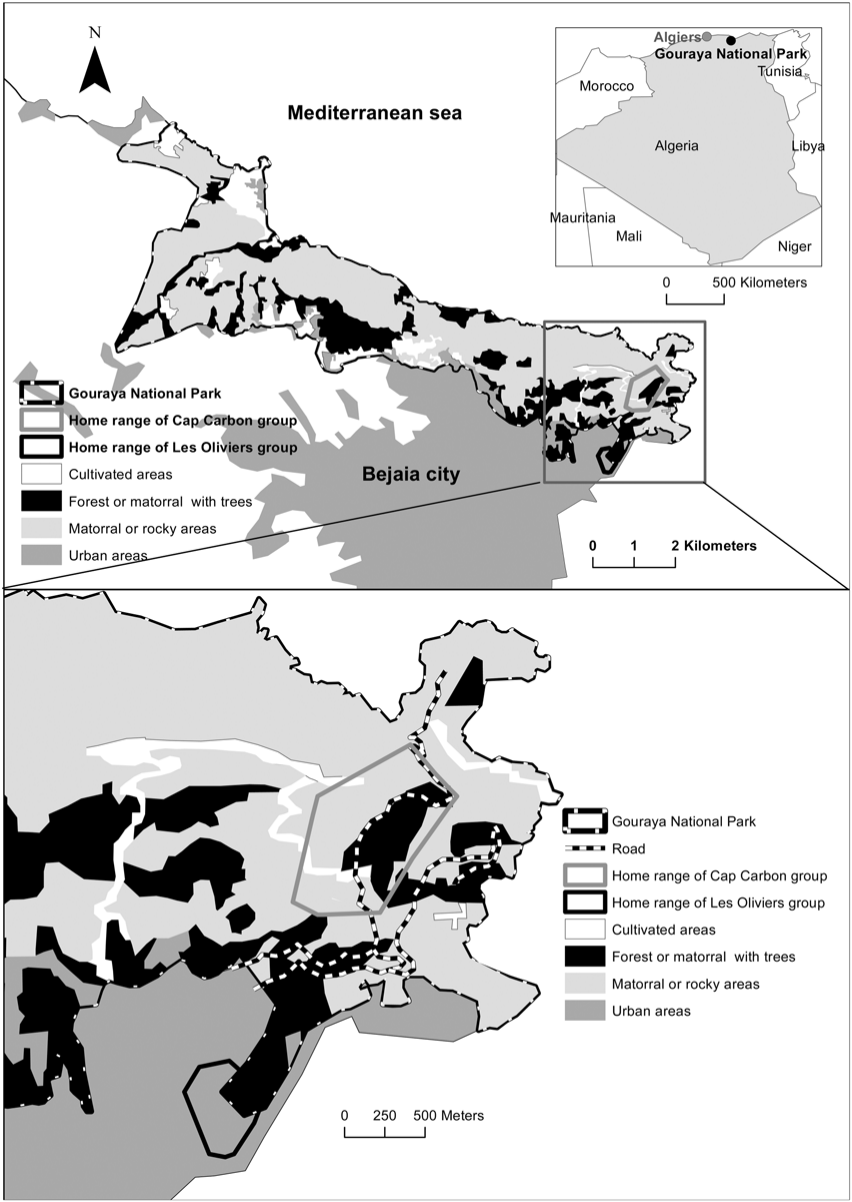
Location of the study site and of the two “Les Oliviers” (peri-urban) and “Cap Carbon” (non-urban) groups.

The study site was located in Gouraya National Park, in eastern Algeria (05° 06’E, 36°46’N, Fig. 1). Gouraya National Park (2,080 ha) is bordered by the Mediterranean Sea to the north and the city of Bejaia to the south. The site is characterized by mild, rainy winters and hot, dry summers. Mean annual rainfall is 968mm and mean monthly temperatures vary from 11.8°C in January to 25.2°C in August. It is a UNESCO Biosphere Reserve and, due to its endemic and rare flora, it is one of the 22 Important Plant Areas (IPAs) in Algeria that have been identified as priority sites for conservation [46,47]. Most forested areas are located in the eastern part of the Park and are dominated by *Pinus halepensis* (Fig. 1). The rest of the park is mainly composed of large matorral shrubland of *Quercus coccifera*, *Ampelodesmos mauritanica* and *Calicotome spinosa* and sub-littoral calcareous cliffs dominated by *Bupleurum fruticosum* and *Euphorbia dendroides* [48].

A small road crosses the eastern part of the Park (Fig. 1). Gouraya Park is a tourist attraction. In particular, the eastern part provides several recreational sites along the road, so that most macaque groups are in frequent contact with visitors. Although feeding the macaques is prohibited, most groups receive food from visitors. We selected two groups (Fig. 1), whose home ranges differed in terms of proximity to urban areas, and that exploited native vegetation or more artificial habitats (e.g. exotic plantations, gardens). The first one, “Les Oliviers” (peri-urban group hereafter), occupied a site located 75 m above sea level, in the immediate vicinity of Bejaia. The second one, “Cap Carbon” (non-urban group hereafter), lived 345 m above sea level, approximately 2 km from Bejaia by road. An artificial mixed forest of *Eucalyptus* sp., *Casuarina* sp., *P*. *halepensis*, *Olea europea*, *Ceratonia siliqua* and *Robinia pseudoacacia* covers most of the home range of the peri-urban group. The home range of the non-urban group is located amid native vegetation described as mainly composed of high matorral shrubland of *P*. *halepensis*-*Q*. *coccifera* and a coastal vegetation dominated by *Bupleurum fruticosum* and *Euphorbia dendroides* [48].

We made our observations between April 2007 and March 2008. The peri-urban and non-urban groups were composed of 31–34 and 40–46 individuals, respectively. We defined age and sex classes as follows: adult males and females (macaques 5+ years old); immatures (individuals 1–4 years old). We did not include infants (individuals < 1 year that were dependant on their mothers and were not weaned) [49] in dietary analyses.

### Data collection and analyses

We noted the phenology (young leaves, mature leaves, flowers, fruit, seeds) of all plant species available to the macaques in the two sites on a monthly basis over the observation periods of the macaques. We recorded feeding activities using an instantaneous scan sampling procedure [50] at 15-min intervals. During each scan sampling, the observer recorded the activity of five randomly chosen individuals, observed from a right to left direction to avoid activity or food item bias. We considered feeding activities only (foraging excluded), *i*.*e*. the picking and actual eating of food items. We noted food names (plant species, other categories of food such as different animals or food from humans, and water) and the vegetative parts of plants (leave, fruit, seed, flower, root, bark). We conducted observations between dawn and dusk for 95 and 88 days (6–10 days *per* group every month) in the peri-urban and non-urban groups, respectively. We performed a total of 3,765 and 2,845 scans in the peri-urban and non-urban groups, totalling about 1,077 and 939 hours of observations, respectively (see details in S1 and S2 Tables). We conducted observations for 56 to 130 hours per month (monthly variation was mainly due to variation in day length over the year, up to five hours). The same researcher, sometimes assisted by a second one, conducted observations. Animals were well habituated to the observer (3–10m close) and visibility was good at every site.

We expressed monthly dietary composition as the percentage of the feeding time macaques spent on various food items, with each group’s monthly diet equal to the mean of diets of the three age-class categories of individuals (adult females, adult males, immatures). We calculated mean annual diet as the average value of all monthly diets. We identified plant species according to a flora [51,52].

We calculated Simpson indices of monthly and annual specific diet diversity (*D*), such as:

$$D={\left(\sum_{i}^{N}p_{k}{}^{2}\right)}^{-1}$$

where *p* is the frequency of different species eaten by macaques. We used an analysis of variance (*anova* function in R) [53] to compare annual specific diversity between groups, using monthly values as replicates.

The observed number of plant species in the diet depends on sample size, so we standardized our estimations of species richness to perform valid comparisons between the two groups. We constructed sample-based rarefaction curves, plotting randomized richness as estimated by the Mao Tau richness estimator with EstimateS [54], against sampling effort, *i*.*e*. the number of hours of observations. Rarefaction generated the expected number of plant species eaten in sets of *n* sampled hours of observations (851 and 737 in the peri-urban and non-urban groups, respectively) with 95% confidence intervals. We then performed statistical comparisons of average species richness between groups using Monte Carlo randomization tests [55]. We used the *rich* package (function *c2m*) in R software [53]. We then plotted the randomized number of species eaten as a function of the number of eaten food items and sampled hours. We also approximated true species richness by computing Chao2 estimators with EstimateS [54]. The Chao2 estimator is useful for extrapolating the expected total species pool using the frequency of rarely sampled species from a series of sampling units to estimate the number of never sampled species [56].

We calculated proportional food overlap between the diets of the two groups using Schoener’s dietary overlap index [57]:

$$C_{\mathrm{Peri}-\mathrm{urban}-\mathrm{Non}-\mathrm{urban}}=1-0.5\sum_{i}^{n}\left(\left|P_{\mathrm{Peri}-\mathrm{urban}.i}-P_{\mathrm{Non}-\mathrm{urban}.i}\right|\right)$$

where *P*_*Peri-urban*.*i*_ and *P*_*Non-urban*.*i*_ are the proportions of food category i (plant species, animals, or human foods) found in diets of the peri-urban and non-urban groups (based on % of feeding time). The index ranges from 0 (no species overlap) to 1 (all food items in equal proportions), with values above 0.6 usually considered to be indicative of significant overlap [58].

We used MANOVA (Wilks test) to investigate differences in percentages of time spent feeding on 11 food categories among months, sites, and macaque age and sex categories. Food categories were human foods, leaves, seeds, acorns, fruit, roots, mushrooms, flowers, animals, bark, water. We transformed our data, which consisted of proportions, to arcsine square roots prior to analyses, which allowed us to normalize proportions and relax the restriction induced by having to sum all variables to 1. We used Bonferroni corrections in cases of multiple comparisons. We made calculations with R software [53]. Additionally, to assess discrimination efficiency among different age-sex categories of individuals, we carried out a multivariate analysis of data by discriminant function analysis, associated with partial Wilk’s lambda tests for each comparison (adult male-adult female, adult male-immature, adult female-immature) (R ADE4package) [53].

## Results

### Phenology

**Fig 2:**
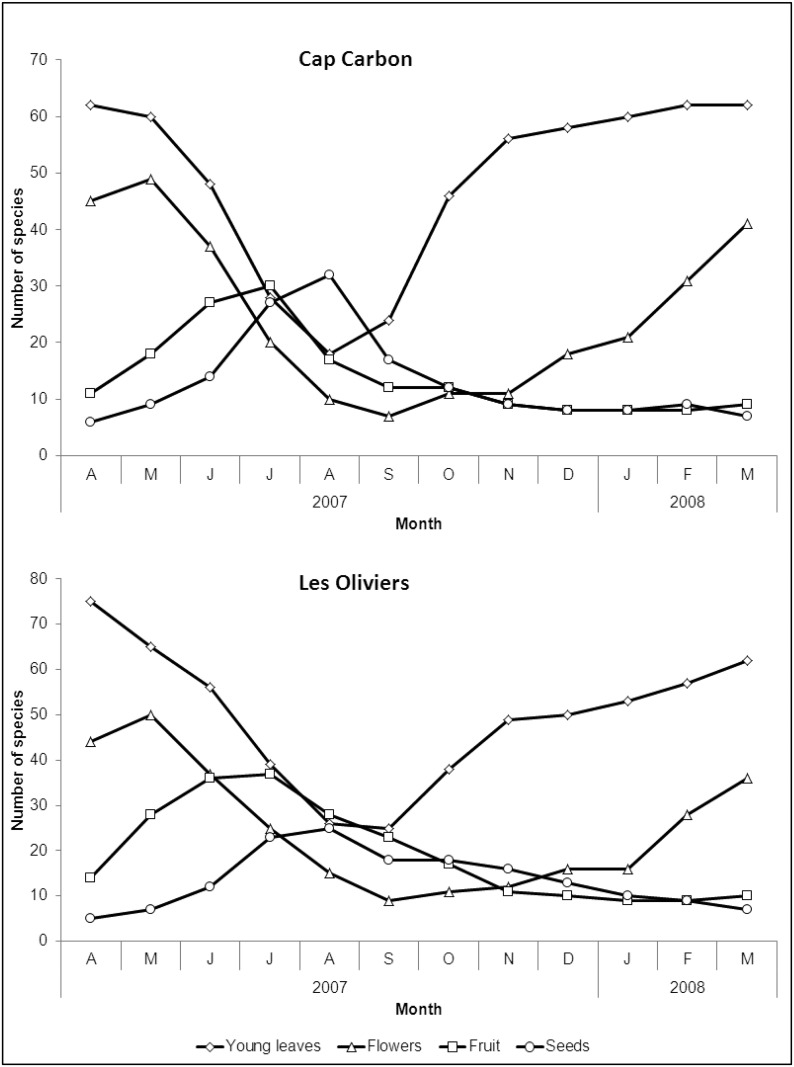
Number of available plant species in each phenological stage at “Les Oliviers” (peri-urban) and “Cap Carbon” (non-urban) sites.

Altogether, we recorded the phenology of 117 plant species in the two sites (77 at the peri-urban site and 66 at the non-urban site). The proportions of exotic species over recorded species, were 19% and 0% at the peri-urban and non-urban sites, respectively (see details in S1 and S2 Tables). At both sites, the pattern of phenology indicated a similar degree of seasonality (Fig. 2, see details in S1 and S2 Tables). For most plant species, budburst started in October and ended in June, and there were 40–75 species available. Growth of vegetation was slower from July to September, although young leaves from 18–39 species were still available at that time. Flowering was at a maximum from February to June, with 28–50 species involved, whereas it was restricted to summer and fall for a few other species (*e*.*g*. *C*. *siliqua*, the lianas *Clematis sp*.). Thus, flowering never stopped totally at any time. Fruit and seeds were available year round from at least five species, with a peak in June-July for fruit and in July-August for seeds (27–37 and 23–32 species, respectively). These results suggest an overall pattern of low seasonality of plant production because none of the categories of plant parts was ever totally disrupted.

### Dietary composition

**Fig 3:**
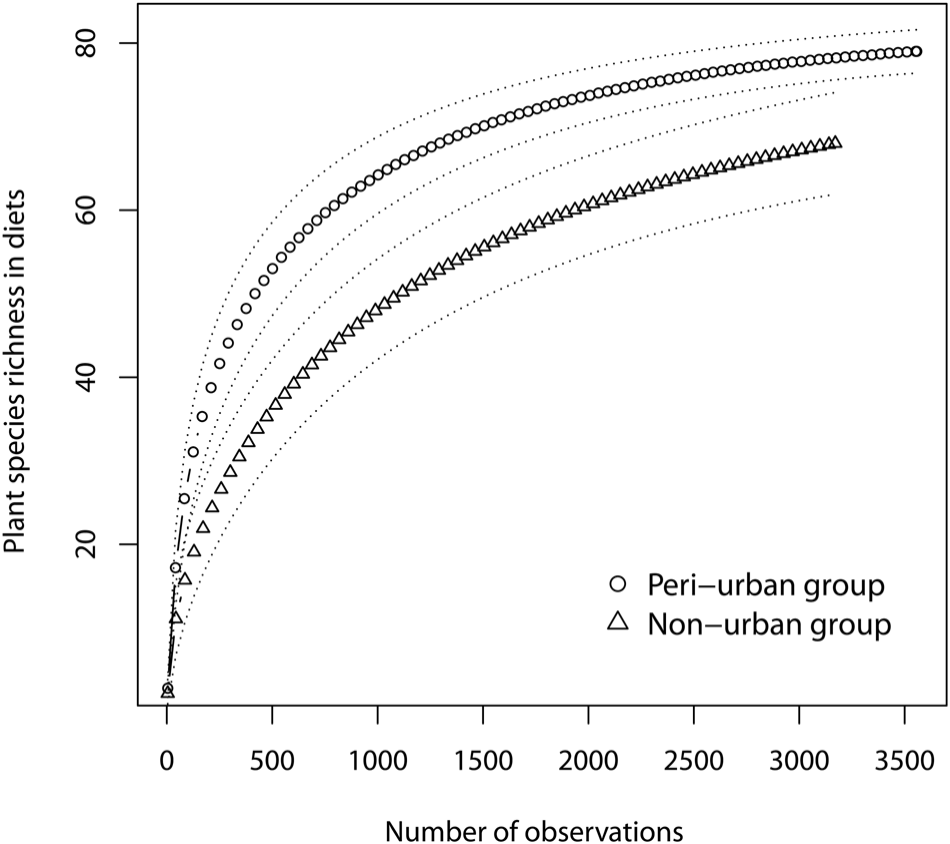
Plant species richness of the macaques’ diets in the two sites. Rarefaction curves represent the cumulative numbers of observed eaten species. Randomized species richness is represented as a function of the number of eaten food items we sampled. Symbols are plotted every 10 hours of observation. Dashed lines indicate 95% confidence intervals.

**Fig 4:**
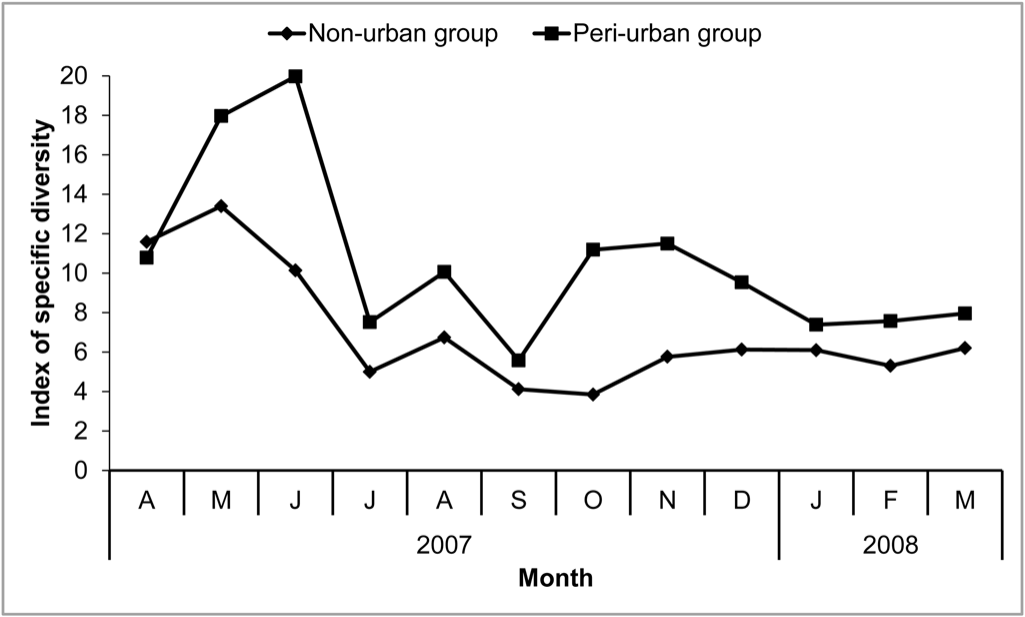
Monthly variation in the specific diversity of the macaques’ diets in the two peri-urban and non-urban groups.

**Dietary species richness and diversity.** We recorded a total of 4,864 (187–936 depending on the month) and 4,441 (142–891 depending on the month) feeding observations from the peri-urban and non-urban groups, respectively. Macaques at Gouraya foraged on a total of 113 plant species (77 and 66 at the peri-urban and non-urban sites, respectively; S1 and S2 Tables). They extracted most of these plants from the herbaceous layer (Table 1). The number of plant species eaten *per* month ranged between 9 and 33 in the non-urban group and between 18 and 55 in the peri-urban group. The rarefaction curve of plant species richness in macaques’ diets almost reached a plateau for both groups (Fig. 3), consistent with true diet richness based on the Chao2 procedure, estimated at 80 and 77 species for the peri-urban and non-urban groups, respectively. We compared species richness from a standardized number of hours of observations (N = 737), and observed higher species richness of diets in the peri-urban group than in the non-urban group (Monte Carlo randomization tests: P < 0.01). Annual diversity of species was greater in the peri-urban group (13.8) than in the non-urban group (10.4, F = 5.51, df = 1, P < 0.05, S1 and S2 Tables), as was monthly diversity of species (except in April, Fig. 4). Dietary breadth was greatest in spring at both sites, concomitantly with plant growth. It was lowest at the end of summer, and slightly increased again in autumn when plants resumed their growth following autumn rains.

**Table 1:** Number of plant species in the macaques’ diet and contribution (%) of each vegetation layer to the diet at each site.

Sites	Cap Carbon (non-urban)	Les Oliviers (peri-urban)
**Number of plant species in the diet**	65	77
**Tree layer**	3	12
**Shrub layer**	18	16
**Herbaceous layer**	39	42
**Lianas**	5	7
**Other categories**	8	6
**Contribution (%) to the diet** ^**a**^		
**Tree layer**	38.3	39.7
**Leaves**	2.7	13.7
**Flowers**	3.8	1.3
**Fruit**	8.5	9.1
**Seeds**	23.0	12.6
**Bark**	0.3	3.0
**Shrub layer**	20.8	5.9
**Leaves**	5.6	3.1
**Flowers**	2.7	0.2
**Fruit**	3.7	2.3
**Seeds & acorns**	1.8 & 7.0	0.3
**Herbaceous layer**	14.2	25.5
**Leaves**	11.7	20.2
**Flowers**	0.5	1.3
**Fruit**	0.7	0.5
**Seeds**	0.1	0.2
**Roots & mushrooms**	0.2 & 1.0	3.2 & 0.1
**Lianas**	5.8	2.0
**Leaves**	5.4	1.7
**Flowers**	0.1	0.1
**Fruit**	0.3	0.2

^a^: the remaining part of the diet was composed of animals, water and human food (see S1 and S2 Tables).

**Fig 5:**
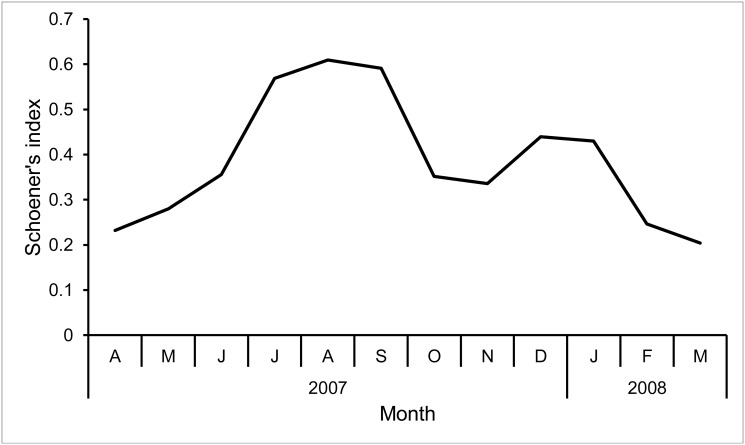
Overlap of the macaque groups’ diets. Monthly variation of Schoener’s indices.

**Dietary overlap.** Schoener’s index revealed low annual (0.44) and monthly dietary overlap between groups (Fig. 5). Only 40 food categories (*i*.*e*. plant species, various invertebrates, and human foods) were common to the two groups (47–54%) while 44 categories belonged to the diet of the peri-urban group only and 35 categories to the diet of the non-urban group only (S1 and S2 Tables). Four (*O*. *europaea*, *P*. *halepensis*, *Fraxinus sp*., *Oxalis pes-capraea*) and three species (*O*. *europaea*, *P*. *halepensis*, *Q*. *coccifera)* accounted for more than 50% of the annual diet of the peri-urban and non-urban groups, respectively.

**Dietary composition.** At both sites, macaques spent most of their mean annual feeding activities eating products from the tree layer (>38%, Table 1). Compared to the non-urban group, the peri-urban group spent one third of the amount of its feeding activities in the shrub layer (21% *vs*. 6%) but twice as much of its feeding time on the herbaceous layer (25% *vs*. 13%, Table 1). Macaques ate various parts of plants, including the bark of five tree species, though in small proportions (S1 and S2 Tables). They spent 80 (peri-urban group) and 86% (non-urban group) of their feeding time collecting natural resources, and spent the rest of their feeding time getting human foods provided by tourists. In addition, the peri-urban group spent 5.2% of its feeding time on the fruit, leaves and flowers of fifteen exotic plant species harvested in domestic or public gardens, while non-urban macaques did not (S1 and S2 Tables).

**Fig 6:**
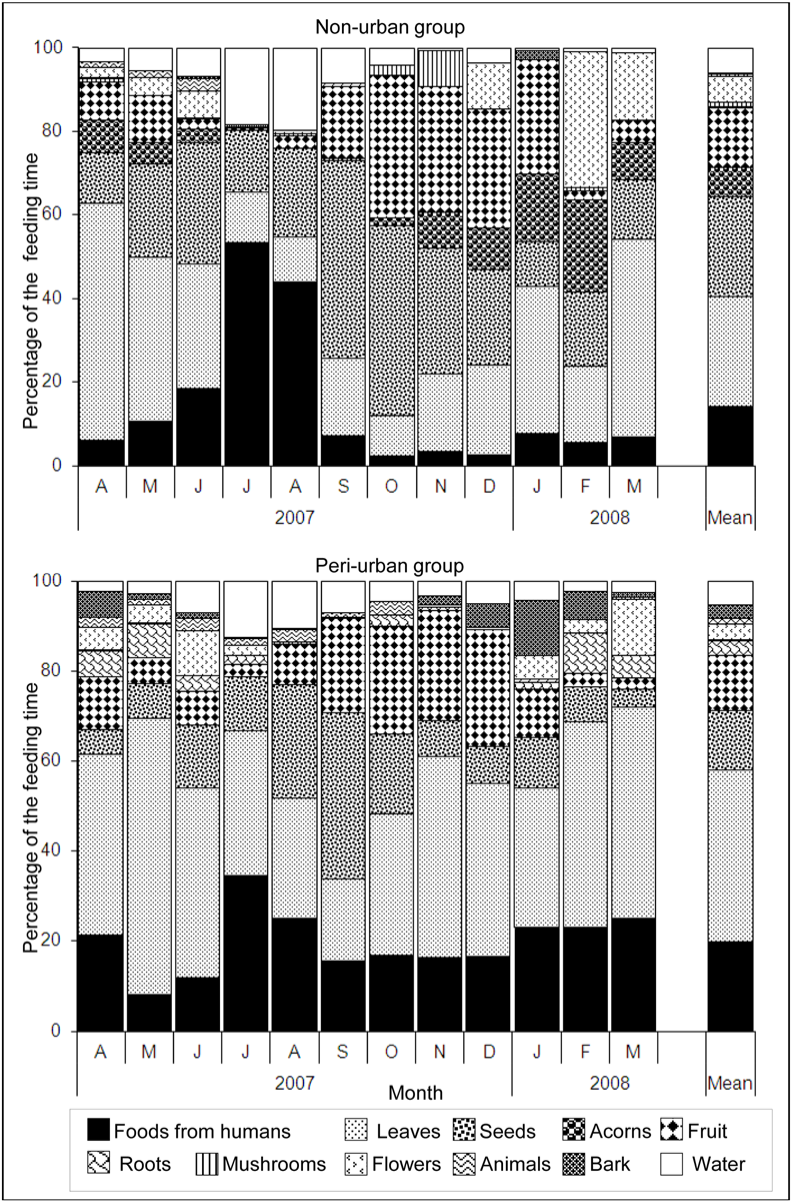
Variation in the percentage of feeding time spent on 11 food categories according to month and group. Leaves also included stems.

Leaves and seeds represented the staple food categories of the macaques’ annual diets (>50% of their feeding time) while twice as many seeds were included in the diet of the non-urban group (seeds + acorns: 31%) compared to the peri-urban group (13%, Fig. 6). The third most consumed food item was fruit (12% for the peri-urban group; 14% for the non-urban group). Most fruit and seeds came from the tree layer (22% for the peri-urban group; 31% for the non-urban group), while leaves came from the herbaceous layer (20% for the peri-urban group; 12% for the non-urban group, Table 1). Underground resources (roots and mushrooms) composed a small proportion of the diet (annual mean: 1.2–3.3% depending on the site, Table 1; up to 9% monthly at both sites, Fig. 6)

**Variation in macaques’ diet.** We showed significant differences among months, groups, age-sex categories of individuals and the month:group interaction (MANOVA, Wilks test, month: *F =* 10.44, *df* = 11, *P <* 0.001; group: *F =* 38.60, *df* = 1, *P <* 0.001; age-sex category: *F =* 7.11, *df* = 2, *P <* 0.001; month:group interaction: *F =* 6.46, *df* = 11, *P <* 0.001). All other interactions were non significant (*P* > 0.05). Variation in macaques’ diet was mainly explained by sharp changes in the proportions of all food items (Table 2). The month:group interaction indicated that monthly variations in the proportions of different foods differed between groups as follows. Leaves were a staple food at both sites in spring (March to May), which coincided with regrowth for most species. Their consumption by the peri-urban group remained high most months, whereas their consumption by the non-urban group decreased sharply from July to February (Fig. 6). At both sites, macaques ate fruit and seeds throughout the year. However, they ate more of them in late summer and autumn, even though few fruiting species were available at that time. These fruit and seeds came mainly from two or three dominant native species, *O*. *europaeus* and *P*. *halepensis* (21–51%) at the peri-urban site and *O*. *europaeus*, *P*. *halepensis*, and *Q*. *coccifera* (52–65% of the feeding time) at the non-urban site. The greatest differences between groups concerned leaf, animal and human food consumption (Table 2). Peri-urban macaques ate more leaves than non-urban macaques did. Although animals (mainly insects) represented a small part of the diets, peri-urban macaques ingested three times as many animals (1.3%) as non-urban macaques (0.4%). Consumption of human foods peaked in July-August at both sites (25–35% at the peri-urban site; 44–53% at the non-urban site, Fig. 6). However, while it remained high at the peri-urban site the other months (17.8 ± 5.4%) it was low at the non-urban site (7.1 ± 4.7%, Fig. 6). People provided a large variety of food items such as fruits, peanuts and starchy foods (bread, pizza and cake, S1 and S2 Tables). Macaques drank water the most in July and August at both sites. Three natural springs were available in the home range of the peri-urban group, while non-urban macaques mainly drank water stored in tree holes or drank from water puddles on rocks.

**Table 2:** Statistical analysis of monthly, inter-group and inter-age-sex category variations in the macaques’ diets.

Food items	*F*_25,46_	*R²*_*adj*_	*P* value	Inter-group *P* value	Inter-category	*P* value
**Leaves**	20.45	0.873	**< 0.0001**	**< 0.0001**	AF-AM	**0.006**
					AF-IM	**< 0.0001**
					AM-IM	**0.010**
**Seeds**	9.09	0.740	**< 0.0001**	0.366	AF-AM	**0.026**
					AF-IM	0.999
					AM-IM	**0.026**
**Fruit**	18.38	0.859	**< 0.0001**	0.138	AF-AM	0.514
					AF-IM	0.187
					AM-IM	0.051
**Acorns**	37.05	0.927	**< 0.0001**	1.000	AF-AM	**0.036**
					AF-IM	0.464
					AM-IM	**0.005**
**Flowers**	13.02	0.809	**< 0.0001**	0.459	AF-AM	0.724
					AF-IM	**0.009**
					AM-IM	**0.003**
**Roots**	9.73	0.755	**< 0.0001**	0.411	AF-AM	0.106
					AF-IM	0.466
					AM-IM	0.364
**Mushrooms**	17.7	0.855	**< 0.0001**	1.000	AF-AM	0.577
					AF-IM	0.318
					AM-IM	0.120
**Bark**	7.61	0.699	**< 0.0001**	0.653	AF-AM	0.743
					AF-IM	**0.0008**
					AM-IM	**0.002**
**Animals**	6.51	0.660	**< 0.0001**	**< 0.0001**	AF-AM	0.178
					AF-IM	0.624
					AM-IM	0.069
**Human foods**	25.29	0.895	**< 0.0001**	**0.0001**	AF-AM	**< 0.0001**
					AF-IM	0.787
					AM-IM	**< 0.0001**

Results of the MANOVA analysis of the “age-sex category + month + group + month*group” model and *P* value composition according to group and age-sex category.

Significant variations are in bold. AM: adult male; AF: adult female; IM: immature.

**Fig 7:**
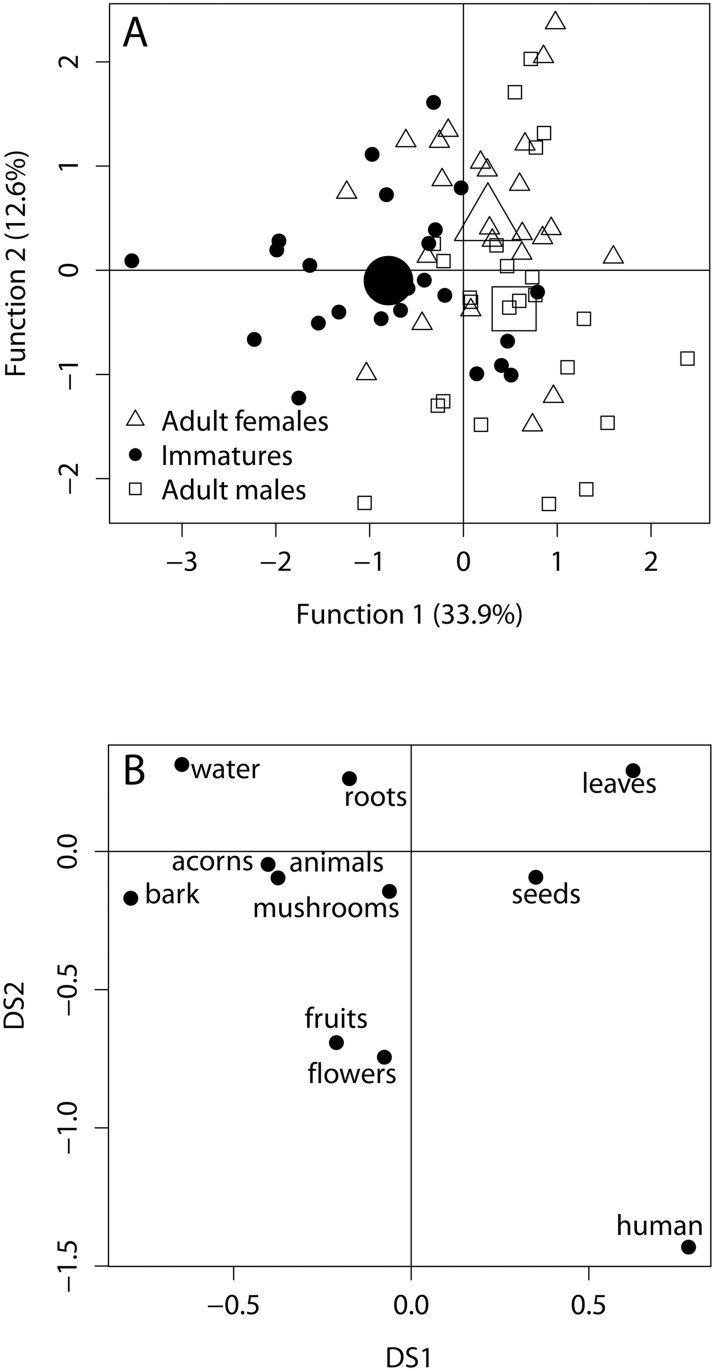
Scatter plots of the discriminant function analysis among three age-sex categories of individuals. A: Scatter plot of two discrimination functions based on samples from adult males, adult females and immatures. The largest symbols indicate the mean of the cloud for each age-sex category; B: Scatter plot of the canonical weight of the discriminant analysis. Variables are the 11 food categories of macaques’ diets.

**Influence of age-sex category.** We computed two different discriminant functions with accounted variability values of 33.9% and 12.6%, respectively. As shown in Fig. 7A, we achieved a good separation of the three categories of individuals by projecting data points onto the space of the first two discriminant functions (MANOVA, Wilks tests, adult male-adult female: *F =* 0.13, *df* = 1, *P <* 0.001; adult male-immature: *F =* 0.14, *df* = 1, *P <* 0.001; adult female-immature: *F =* 0.13, *df* = 1, *P <* 0.001). Human foods were the food items that best discriminated between age-sex categories (see also Table 2). Adult males exploited more human foods than adult females and immatures did (Fig. 7B). Adults females ate more leaves than immatures and adult males did (Fig. 7B). Immatures ate more flowers and more bark than adults and more acorns than adult males did (Fig. 7B). The other food items did not allow for discriminating between age-sex categories (Table 2).

**Summary**. Our results suggest a low seasonality of natural resource availability at Gouraya because none of the plant part categories suffered from seasonal shortage of its production. Macaques’ diets varied monthly. Nevertheless, we noticed that leaves, seeds, and fruit continuously composed the diets year round, although in various proportions. Most of the numerous consumed species came from the herbaceous layer. However, macaques spent a great proportion of their mean annual feeding time on tree products. They were mainly leaf- and seed-eaters, while their diets relied on subterranean resources in a small proportion. The proximity of urban areas led the macaques to exploit human foods, all the more so as macaques were closer to the city. Human food consumption peaked in the summer months, while it remained at a high level for the group closer to the city compared to the farther group the rest of the year. Species richness and diversity of macaques’ diets were the highest in close proximity to the city, partly because of the availability of exotic plant species. Among macaques, adult males were those that most exploited human foods, while adult females relied more on leaves, and immatures relied more on flowers, acorns and bark.

## Discussion

### Barbary macaque diet flexibility in a relictual habitat

Populations living at the edge of a species’ range or in relictual or marginal habitats have been under focus because they allow us to investigate the evolutionary potential of species and their ecological plasticity when they have to cope with changing environments [59–62].

Cedar-oak forests, which host most of the existing wild populations of Barbary macaques, were initially suggested to be their preferred habitat [38]. However, the past history of populations and habitat alteration by humans best explain their modern distribution [63]. Gouraya habitats are relictual and represent less than 0.6% of the species range at the eastern edge (calculated from data drawn from [63] and [43]). In Gouraya, macaques were exposed to relatively less severe seasonal constraints of natural resource availability compared to other habitats (cedar-oak or deciduous oak forests) [64]. This is linked to a sub-humid temperate climate and the proximity of the sea that produces dense, low clouds and mist, which contributes to mild winters and mitigates the effects of the summer drought on the vegetation. In Gouraya, Winter mean monthly temperatures are never below zero, contrary to mountain forests [65]. Because of these particular conditions, in our study Barbary macaques consumed fruit and seeds at different rates every month, while in other habitats they do not eat fruit and seeds for several months on end because of seasonal shortage. Dietary species diversity in Gouraya was similar to that in other habitats [40,66], and dietary species richness was in the range of mountainous forests (deciduous oak, cedar-oak, or fir forests) [40,41,66]. The low degree of dietary overlap between the two groups likely reflects the mosaic of the plant cover at Gouraya Park, although the two groups’ diets primarily relied on the same two dominant species, *O*. *europaea* and *P*. *halepensis*. Both groups at Gouraya relied on the herbaceous layer to a lesser extent than in other sites (half as much as in deciduous oak forest, and three times less than in cedar-oak forest) [40,66] while they collected nearly half of their diet from trees, shrubs, or lianas. Leaves were a staple food as in other habitat types [40,66], while seeds and fruit represented a greater amount of the diet at Gouraya than in other habitats [40,66]. Barbary macaques at Gouraya relied little on subterranean resources whatever the month, unlike Barbary macaques in other habitats, which consume large amounts of roots, mushrooms or subterranean invertebrates in the dry months of food scarcity [40,66], or where competition with domestic livestock is strong [39]. Those food items are generally difficult to excavate, time-consuming and costly [67].

In conclusion, compared to macaques living in habitats of the major part of the distribution area, Gouraya macaques are subjected to less severe seasonal constraints, they consume a greater amount of fruit and seeds that are available much of the year, thanks to an even temporal distribution of tree production, and their diet contains more accessible (and less costly) foods than subterranean foods. Although we do not know whether a high nutritional value can compensate for the costs induced by foraging subterranean foods or not, these results suggest that most current populations live in harsher environments than macaques at Gouraya Park, and that Gouraya vegetation does not represent a marginal habitat for the species. The expansion of the Gouraya population, associated with habitat restoration and protection, appears to support this statement. This population estimated to be composed of about 50 individuals 30 years ago, mainly localised in the upper part of the site [38], has expanded as far as Bejaia city. Our results improve our knowledge about the diet flexibility of the species and about its ability to adapt to various habitats. The results underline the need to distinguish between geographic peripheral position and ecological marginality in Barbary macaque habitats.

### Effects of peri-urban colonization

The group near the city benefited from daily visits by local people who travel on foot; conversely, tourists reached the farthest sites mainly by car, so that interactions with macaque groups were mainly restricted to the summer holiday period. Consequently the two groups’ diets varied depending on their distance from the city. Peri-urban macaques ate more human foods, easily accessible and of high energy content, than farther macaques did, and did so throughout the year. Their diets included higher species richness in food resources, partly due to human foods and exotic plant species availability. This could be a positive factor for macaque population growth and expansion.

Adult male Barbary macaques ate a greater proportion of human foods than adult females and immatures did, which suggests that they have a priority access to these highly energetic foods and/or that they are less fearful of humans. This is in accordance with observations in the provisioned population of Gibraltar where adult males were more prone to interact with tourists than adult females or immatures [68] and with observations on chacma baboons where dominant males had priority of access to urban food sources [69]. In non-provisioned populations, adult males and adult females are mainly leaf-eaters, while immatures consume a greater proportion of seeds, including acorns [40,66]. This suggests that adaptation to urban zones could be mainly driven by changes in male behaviour. Because adult males are the initiators of group movements [70], human feeding macaques may cause groups to stay longer at these sites, and in doing so over-exploit some parts of the natural vegetation of their home ranges. As a consequence, they might become even more dependent on human foods.

Barbary macaques may suffer the negative effects of urban life. The high energy content of anthropogenic food may have a noticeable negative effect on their health, e.g., leading to obesity (S1 Appendix). Like other urban monkeys [71], Barbary macaques often use electric wires for travelling, and some are killed by electrocution (S1 Appendix). Macaques will raid people’s crops and forage in their garbage (S1 Appendix), which causes damage to property and stress for local residents, who sometimes respond with aggression, *e*.*g*., poisoning or slingshot shooting (S1 Appendix). Nevertheless, quantifying those effects and estimating to what extent they negatively affect the demography of urban groups remains to be done.

### Conservation implications

For Barbary macaques, Gouraya habitats appear at least as suitable as mountain forests. The expansion of the population as far as Bejaia city suggests efficient effects of habitat conservation actions. However, this population is located on a small area, so it remains sensitive to extinction risks.

Defining good urban exploiters is tricky. For instance, among carnivores, animals that have generalist habitat requirements, are crepuscular or nocturnal, show social flexibility, or have wide home ranges seem to be the best cities exploiters [72,73]. Apart from some primates [24,31,74–76], diurnal social mammals living in large cohesive groups likely to frequently clash with humans seem less adapted for residence in urban environments.

Barbary macaques are large diurnal mammals that live in large multi-male-multi-female groups of up to 88 individuals, on small stable home ranges [77]. In addition, unlike other non-human primates that have colonized urban zones [24,31,75,76], the Barbary macaque is a non-weed species [37]: it avoids crossing wide open areas, because it is a habitat-specialist that depends on forest or cliffs for protection from danger [38,65,78]. These socio-ecological features should have deterred Barbary macaques from invading urban zones. However, four simultaneously acting factors can explain why they colonized the peri-urban areas of Bejaia city: (i) the gradual restoration of now actively protected forested habitats has allowed for macaque population expansion, (ii) the expanding city has come into contact with macaques’ habitats, (iii) the high diet flexibility of the species, and (iv) by feeding macaques (even in the upper part of the site), tourists reduce the macaques’ fear of humans. The peri-urban group is one of the groups located at the colonization front of the population towards the city.

Studies of the colonization processes of cities by wildlife and related influential factors are scarce and mainly concern birds [12,79,80]. It is then highly critical to keep monitoring the dynamics of the Gouraya population at the colonization front. Provisioning may cause a higher growth rate of the population and/or higher fecundity of group members, which could increase the probability for the population to expand towards the city center. Urban zones may be an ecological trap for Barbary macaques if changes, either in their structure or in human behaviour [81–83], induce negative demographic effects for urban exploiter macaques. For instance, humans may consider them as pests, and injure or kill them, because they damage property [84] or they (especially adult males) directly attack humans to obtain food, as also observed in other macaques [75]. Close contact with humans, often with direct human-monkey hand contacts, can favour parasite transmission, as observed in other provisioned macaques [85]. In addition, wild populations that have a diminished fear of humans may be more likely to be poached (S1 Appendix). This is the case in Morocco, where Barbary macaque infants of groups living close to tourist sites are prime targets for poachers [86]. As observed in other studies [87], consequences for Barbary macaques could be reduced fitness (in particular lower survival), and an increased extinction risk compared to non-urban habitats. As conflicts between humans and many mammal species are often costly challenges for wildlife managers [21,22], we stress that it is critical to anticipate dramatic human/Barbary macaque conflicts. At Gouraya, managers should consider the rehabilitation of macaques back to their natural habitat to prevent colonization of the city center by macaques over the years. Because those macaques usually avoid crossing open areas, environment and urban management should maintain open areas as buffer zones between macaques’ habitats and the urban domain. Restricting anthropogenic food resources from waste sites and human provisioning may address the issue related to the human-macaque interface. Future management actions should be based on knowledge not only about macaque ecology studies, but also about sociological ones [76,88,89].

## Supporting Information

S1 Table Mean annual and monthly variations in the diet of the peri-urban Barbary macaque group ‘Les Oliviers’ at Gouraya National Park (Algeria) and phenology of food items. (DOCX)

S2 Table Mean annual and monthly variations in the diet of the non-urban Barbary macaque group ‘Cap Carbon’ at Gouraya National Park (Algeria), and phenology of food items. (DOCX)

S3 Table Proportions of the monthly feeding times spent on 11 food categories by Barbary macaques from the three age-sex categories at the Cap Carbon (non-urban) and Les Oliviers (peri-urban) sites. AM: adult male; AF: adult female; IM: immature.
(XLSX)

S1 Appendix Negative effects of urban colonization by Barbary macaques on human property and on Barbary macaques. (DOCX)
