# Supplementary material for: Is Diet Flexibility an Adaptive Life Trait for Relictual and Peri-Urban Populations of the Endangered Primate Macaca sylvanus?
Source: PLoS One. 2015 Feb 25;10(2):e0118596. doi: 10.1371/journal.pone.0118596 (PMC4340959; doi:10.1371/journal.pone.0118596)
Supplement: S1 Table — (DOCX) [file pone.0118596.s001.docx]

Table S1. Mean annual and monthly variations in the diet of the peri-urban Barbary macaque group ‘Les Oliviers’ at Gouraya National Park (Algeria) and phenology of food items.

|  |  |  |  | Years | 2007 |  |  |  |  |  |  | |  | |  | | 2008 | |  | |  |
| --- | --- | --- | --- | --- | --- | --- | --- | --- | --- | --- | --- | --- | --- | --- | --- | --- | --- | --- | --- | --- | --- |
|  | Family | Species | Plant part | Mean annual | A | M | J | J | A | S | O | | N | | D | | J | | F | | M |
| Tree layer | Casuarinaceae | *Casuarina equisetifolia** | leave | + |  |  |  |  |  |  |  | |  | |  | | 0.5 | |  | |  |
|  | Cupressaceae | *Cupressus sempervirens* | bark | 0.1 | 0.3 | 0.3 | 0.2 |  |  |  |  | |  | |  | | 0.5 | |  | |  |
|  |  |  | seed | + |  |  |  |  |  | 0.4 |  | |  | |  | |  | |  | |  |
|  | Leguminoseae | *Ceratonia siliqua* | bark | 2.8 | 5.6 | 0.8 | 0.7 | 0.4 |  |  |  | | 2.1 | | 5.2 | | 11.8 | | 6.2 | | 1.0 |
|  |  |  | leave | + |  | 0.1 |  | 0.1 |  |  |  | |  | |  | |  | |  | |  |
|  |  |  | fruit | 0.1 | 0.3 |  | 0.1 | 0.3 |  |  |  | |  | |  | |  | |  | |  |
|  |  |  | seed | 0.4 |  | 0.8 | 3.4 | 0.1 |  |  |  | |  | |  | |  | |  | |  |
|  |  | *Robinia pseudoacacia** | leave | 0.7 | 1.8 | 0.5 | 0.7 | 2.2 | 2.0 | 0.8 | 0.5 | | 0.4 | |  | |  | |  | |  |
|  |  |  | flower | 0.5 | 3.5 | 1.8 | 0.8 | 0.1 |  |  |  | |  | |  | |  | |  | |  |
|  |  | *Gleditsia triacanthos* | leave | 0.1 |  | 0.4 |  | 0.4 |  |  |  | |  | |  | |  | |  | |  |
|  |  |  | fruit | 1.9 |  |  |  |  |  | 4.0 | 5.1 | | 8 | | 5.2 | |  | |  | |  |
|  |  | *Cercis siliquastrum* | bark | + |  |  |  |  | 0.2 |  |  | |  | |  | |  | |  | |  |
|  |  |  | leave | 1.8 | 1.2 | 4.6 | 5 | 3.0 | 3.6 | 2.4 | 0.5 | |  | |  | |  | |  | | 1.5 |
|  |  |  | flower | 0.8 | 1.2 |  |  |  |  |  |  | |  | |  | |  | |  | | 8.0 |
|  |  |  | fruit | 0.1 |  | 1.2 | 0.2 |  |  |  |  | |  | |  | |  | |  | |  |
|  |  |  | seed | + |  | 0.4 | 0.1 |  |  |  |  | |  | |  | |  | |  | |  |
|  | Moraceae | *Ficus microcarpa** | fruit | 0.3 |  | 1.2 |  | 0.7 |  |  |  | |  | |  | |  | | 1.2 | |  |
|  |  |  | seed | + |  | 0.5 |  |  |  |  |  | |  | |  | |  | |  | |  |
|  |  | *Ficus carica** | leave | 0.4 | 0.9 | 1.5 | 0.8 | 0.9 |  | 0.4 |  | |  | |  | |  | |  | |  |
|  |  |  | fruit | 0.4 |  |  | 0.2 |  | 2.0 | 0.4 |  | |  | |  | |  | | 1.7 | |  |
|  |  | *Morus alba** | bark | + |  |  | 0.2 |  |  |  |  | |  | |  | |  | |  | |  |
|  |  |  | fruit | 0.3 | 2.1 | 0.8 | 0.2 |  |  |  |  | |  | |  | |  | |  | |  |
|  | Oleaceae | *Fraxinus excelsior* | buds | 0.7 |  |  |  |  |  |  |  | |  | | 6.7 | | 2.1 | |  | |  |
|  |  |  | leave | 6.0 | 12.6 | 3.0 | 2.2 | 3.7 | 2.2 | 0.4 |  | | 5 | | 1.6 | | 1.1 | | 21.2 | | 19.1 |
|  |  |  | fruit | 0.1 |  |  |  |  |  |  | 1.5 | |  | |  | |  | |  | |  |
|  |  | *Olea europaea* | leave | 1.2 | 0.9 | 2.6 | 1.3 | 0.5 | 0.7 |  |  | |  | | 1.6 | | 2.7 | | 2.1 | | 2.0 |
|  |  |  | fruit | 6.0 |  |  |  |  | 1.1 | 14.9 | 11.7 | | 13.4 | | 20.7 | | 10.7 | |  | |  |
|  |  | *Olea europaea* | seed | 5.0 | 6.8 | 5.6 | 8.4 | 6.7 | 4.0 | 1.2 | 3.0 | | 2.1 | | 0.5 | | 10.7 | | 7.5 | | 3.0 |
|  | Pinaceae | *Pinus halepensis* | flower | 0.7 |  |  |  |  |  |  |  | |  | |  | | 5.3 | | 2.5 | |  |
|  |  |  | seed | 7.5 | 0.3 | 1.1 | 1.7 | 4.9 | 18.8 | 34.9 | 12.7 | | 5.9 | | 7.8 | | 0.5 | | 0.4 | | 1.0 |
|  | Ulmaceae | *Celtis australis* | buds | 0.1 |  |  |  |  |  |  |  | |  | |  | |  | |  | | 1.0 |
|  |  |  | leave | 2.4 | 1.2 | 10.5 | 3.3 | 8.7 | 1.1 | 2 | 1.0 | | 0.4 | |  | |  | |  | |  |
|  |  |  | fruit | 0.4 | 0.9 | 0.1 | 0.2 |  |  | 1.2 |  | | 2.1 | |  | |  | |  | |  |
|  |  |  | seed | + |  |  | 0.1 |  |  |  |  | |  | |  | |  | |  | |  |
| Shrub layer | Anacardiaceae | *Pistacia lentiscus* | flower | 0.1 |  |  |  |  |  |  |  | |  | |  | |  | |  | | 1.0 |
|  |  |  | fruit | + |  |  |  |  |  |  |  | | 0.4 | |  | |  | |  | |  |
|  | Asparagaceae | *Asparagus acutifolius* | buds | + |  | 0.1 |  |  |  |  |  | |  | |  | |  | | 0.4 | |  |
|  | Capparaceae | *Capparis spinosa* | fruit | + |  |  |  |  | 0.4 |  |  | |  | |  | |  | |  | |  |
|  | Euphorbiaceae | *Ricinus communis* | leave | + |  | 0.1 |  |  |  |  |  | |  | |  | |  | |  | |  |
|  | Moraceae | *Broussonetia papyrifera** | leave | 0.1 |  | 0.1 | 0.2 |  |  |  |  | | 0.4 | |  | |  | |  | |  |
|  | Leguminoseae | *Acacia karroo* | leave | 1.5 |  | 0.4 | 1.1 | 1.6 | 4.9 | 5.2 | 2.0 | | 2.1 | |  | |  | | 0.4 | | 0.5 |
|  |  |  | flower | + |  |  |  | 0.1 |  |  |  | |  | |  | |  | |  | |  |
|  | Malvaceae | *Hibiscus palustris** | flower | 0.1 |  |  |  | 0.2 |  |  |  | | 0.4 | |  | |  | |  | |  |
|  | Oleaceae | *Jasminum fruticans** | leave | + |  | 0.4 |  |  |  |  |  | |  | |  | |  | |  | |  |
|  |  |  | fruit | + |  |  | 0.1 |  |  |  |  | |  | |  | |  | |  | |  |
|  | Punicaceae | *Punica granatum** | fruit | 0.4 |  |  | 0.9 | 0.6 | 2.9 |  |  | |  | |  | |  | |  | |  |
|  |  |  | seed | 0.2 |  |  |  |  | 1.3 | 0.4 | 1.0 | |  | |  | |  | |  | |  |
|  | Rhamnaceae | *Rhamnus alaternus* | fruit | 0.1 |  |  | 1.3 | 0.4 |  |  |  | |  | |  | |  | |  | |  |
|  |  |  | seed | + |  | 0.1 | 0.4 |  |  |  |  | |  | |  | |  | |  | |  |
|  | Rosaceae | *Rubus ulmifolius* | leave | 0.8 | 0.6 | 1.1 | 0.2 | 0.2 |  | 0.4 |  | 1.3 | | 2.1 | | 0.5 | | 0.4 | | 3 | |
|  |  | *Crataegus azarolus* | leave | 0.1 | 0.3 |  |  |  |  |  |  | |  | |  | | 0.5 | |  | |  |
|  |  |  | fruit | 0.2 |  |  |  |  |  | 0.4 | 1.5 | |  | |  | |  | |  | |  |
|  |  | *Eriobotrya japonica** | flower | + |  |  |  |  |  |  |  | |  | | 0.5 | |  | |  | |  |
|  |  |  | fruit | 0.9 | 6.7 | 1.0 |  |  |  |  |  | |  | |  | |  | |  | | 2.5 |
|  |  | *Rosa sp.** | buds | + |  |  | 0.3 |  |  |  |  | |  | |  | |  | |  | |  |
|  |  |  | stem | 0.1 |  | 0.3 |  | 0.7 |  | 0.4 |  | |  | |  | |  | |  | |  |
|  |  | *Cydonia oblonga** | fruit | 0.3 |  |  |  |  |  |  | 3.6 | |  | |  | |  | |  | |  |
| Lianas | Vitaceae | *Vitis vinifera** | leave | 0.1 |  | 0.1 | 0.6 |  | 0.7 |  |  | |  | |  | |  | |  | |  |
|  |  |  | fruit | 0.2 |  |  |  | 0.2 | 1.6 |  | 0.5 | |  | |  | |  | |  | |  |
|  | Ranunculaceae | *Clematis cirrhosa* | leave | + | 0.3 |  |  |  |  |  |  | |  | |  | |  | |  | |  |
|  |  | *Clematis flammula* | leave | + |  | 0.1 |  |  |  |  |  | |  | |  | |  | |  | |  |
|  |  |  | flower | + |  |  |  | 0.1 |  |  |  | |  | |  | |  | |  | |  |
|  | Sapindaceae | *Cardiospermum sp.* | leave | + |  | 0.1 | 0.2 |  |  |  |  | |  | |  | |  | |  | |  |
|  | Smilacaceae | *Smilax aspera* | leave | 1.1 |  |  | 0.4 | 0.5 | 0.2 | 0.4 | 0 | | 4.2 | | 4.7 | | 1.6 | | 0.8 | | 0.5 |
|  | Tropaeolaceae | *Tropaeolum majus* | leave | 0.2 |  | 0.3 | 0.9 | 0.2 |  |  |  | |  | | 0.5 | |  | | 0.4 | |  |
|  |  |  | flower | 0.1 |  |  | 0.9 |  |  |  |  | |  | |  | |  | |  | |  |
|  |  |  | fruit | + |  |  | 0.3 |  |  |  |  | |  | |  | |  | |  | |  |
|  | Vitaceae | *Parthenocissus quinquefolia** | leave | 0.3 |  |  | 0.1 | 0.6 | 1.8 |  | 0.5 | |  | |  | |  | |  | |  |
| Herbaceous layer | Acanthaceae | *Achanthus mollis* | flower | 0.7 |  |  | 7.2 | 1.6 |  |  |  | |  | |  | |  | |  | |  |
|  |  |  | fruit | + |  |  |  | 0.1 |  |  |  | |  | |  | |  | |  | |  |
|  | Amaranthaceae | *Achyranthes aspera* | leave | 0.7 |  | 0.1 | 1.6 | 0.9 | 0.7 |  | 0.5 | | 1.3 | | 0.5 | | 0.5 | | 0.8 | | 1.5 |
|  | Boraginaceae | *Borago officinalis* | leave | + |  | 0.3 |  |  |  |  |  | |  | |  | |  | |  | |  |
|  | Asteraceae | *Centaurea pullata* | stem | 0.1 |  |  |  |  |  |  |  | |  | |  | |  | | 0.8 | |  |
|  |  |  | flower | + |  | 0.1 | 0.1 |  |  |  |  | |  | |  | |  | |  | |  |
|  |  |  | fruit | + |  |  | 0.2 | 0.1 |  |  |  | |  | |  | |  | |  | |  |
|  |  | *Galactites elegans* | flower | + | 0.3 |  | 0.1 |  |  |  |  | |  | |  | |  | |  | |  |
|  |  |  | fruit | 0.1 |  |  | 0.7 |  |  |  |  | |  | |  | |  | |  | |  |
|  |  | *Calendula arvensis* | leave | + |  | 0.3 | 0.1 |  |  |  |  | |  | |  | |  | |  | |  |
|  |  |  | flower | + |  | 0.4 |  |  |  |  |  | |  | |  | |  | |  | |  |
|  |  | *Pallenis spinosa* | leave | 0.2 |  | 0.4 | 1.4 |  |  |  |  | |  | |  | |  | |  | |  |
|  |  | *Sonchus oleraceus* | leave | 0.1 | 0.3 | 0.4 | 0.2 |  |  |  |  | |  | |  | |  | | 0.4 | |  |
|  | Convolvulaceae | *Convolvulus althaeoides* | leave | 0.2 |  | 0.3 | 0.3 | 0.1 |  |  |  | | 0.4 | |  | |  | | 0.8 | |  |
|  |  |  | flower | 0.1 |  | 0.8 |  |  |  |  |  | |  | |  | |  | |  | |  |
|  |  |  | fruit | + |  |  | 0.2 |  |  |  |  | |  | |  | |  | |  | |  |
|  |  | *Convolvulus sabatius* | leave | 0.1 | 0.3 | 0.1 | 0.2 |  |  |  |  | |  | |  | |  | |  | |  |
|  | Cruciferae | *Sinapis arvensis* | stem | + |  | 0.1 |  |  |  |  |  | |  | |  | |  | |  | |  |
|  |  |  | fruit | + |  |  | 0.1 |  |  |  |  | |  | |  | |  | |  | |  |
|  | Cyperaceae | *Carex divisa* | leave | 0.1 |  | 0.3 | 0.2 |  |  |  |  | |  | |  | |  | | 0.4 | |  |
|  |  |  | flower | + |  | 0.1 | 0.2 |  |  |  |  | |  | |  | |  | |  | |  |
|  | Dioscoreaceae | *Dioscorea communis* | leave | 0.5 |  | 0.1 | 0.8 | 1.6 | 1.6 |  | 2.0 | |  | |  | |  | | 0.4 | |  |
|  | Geraniaceae | *Geranium robertianum* | leave | 0.1 |  |  |  |  |  |  |  | |  | |  | |  | | 0.4 | | 0.5 |
|  |  |  | fruit | + |  | 0.1 |  |  |  |  |  | |  | |  | |  | |  | |  |
|  | Fumariaceae | *Fumaria capreolata* | leave | + |  | 0.3 |  |  |  |  |  | |  | |  | |  | |  | |  |
|  | Malvaceae | *Malva sylvestris* | leave | 1.1 | 2.1 | 4.5 | 0.8 |  |  |  | 1.5 | | 1.7 | | 1.0 | | 1.1 | | 0.4 | | 0.5 |
|  |  |  | fruit | + |  |  |  | 0.1 |  |  |  | |  | |  | |  | |  | |  |
|  |  |  | seed | 0.1 |  |  |  | 0.2 | 1.1 |  |  | |  | |  | |  | |  | |  |
|  | Oxalidaceae | *Oxalis pes-caprae* | leave | 2.4 | 2.6 | 1.9 |  |  |  |  |  | | 7.6 | | 4.7 | | 4.3 | | 4.1 | | 3.0 |
|  |  |  | fruit | + |  |  |  |  |  |  |  | | 0.4 | |  | |  | |  | |  |
|  |  |  | root | 3.2 | 5.9 | 7.3 | 3.7 | 1.9 | 0.4 | 0.4 | 2.5 | | 0.8 | |  | | 1.6 | | 9.1 | | 5.0 |
|  | Fabaceae | *Trifolium sp* | leave | + |  | 0.4 | 0.1 |  |  |  |  | |  | |  | |  | |  | |  |
|  | Plantaginaceae | *Plantago major* | leave | 0.1 |  |  |  |  |  |  |  | |  | |  | |  | | 0.8 | | 0.5 |
|  | Poaceae | *Hyparrhenia hirta* | leave | + |  | 0.1 |  |  |  |  |  | |  | |  | |  | |  | |  |
|  |  | *Catapodium rigidum* | leave | 1.5 | 2.3 | 1.8 | 2 | 0.5 | 1.1 | 0.8 | 0.5 | | 2.1 | | 0.5 | | 3.2 | | 2.1 | | 0.5 |
|  |  |  | root | + |  |  |  | 0.1 |  |  |  | |  | |  | |  | |  | |  |
|  |  |  | flower | 0.2 |  | 0.1 | 0.3 |  |  |  |  | |  | |  | |  | |  | | 2.0 |
|  |  | *Brachypodium sylvaticum* | leave | 1.4 | 2.6 | 1.8 | 0.3 |  |  |  | 4.6 | | 2.9 | | 2.1 | |  | | 0.4 | | 2.5 |
|  |  |  | flower | + |  | 0.1 |  |  |  |  |  | |  | |  | |  | |  | |  |
|  |  | *Cynodon dactylon* | leave | + |  | 0.4 | 0.1 |  |  |  |  | |  | |  | |  | |  | |  |
|  |  | *Hordeum murinum* | leave | 0.1 |  | 0.5 | 0.7 |  |  |  |  | |  | |  | |  | |  | |  |
|  |  |  | flower | + |  |  | 0.1 |  |  |  |  | |  | |  | |  | |  | |  |
|  |  | *Piptatherum miliaceum* | leave | 4.3 | 0.3 | 1.2 | 5.5 | 2.9 | 1.6 | 2.8 | 11.7 | | 11.3 | | 6.7 | | 1.6 | | 4.6 | | 1.5 |
|  |  | *Anisantha rubens* | leave | 0.1 |  | 0.8 |  | 0.1 |  |  |  | |  | |  | |  | |  | |  |
|  |  | *Dactylis glomerata* | leave | + |  | 0.1 |  |  |  |  |  | |  | |  | |  | |  | |  |
|  | Ranunculaceae | *Ranunculus muricatus* | fruit | 0.2 |  |  | 2.4 |  |  |  |  | |  | |  | |  | |  | |  |
|  |  |  | seed | 0.1 |  | 0.7 |  |  |  |  |  | |  | |  | |  | |  | |  |
|  | Alliaceae | *Allium cepa** | stem | 0.1 | 0.6 |  |  |  |  |  |  | |  | |  | |  | |  | |  |
|  |  |  | leave | + |  |  | 0.1 |  |  |  |  | |  | |  | |  | |  | |  |
|  | Solanaceae | *Solanum nigrum* | leave | 0.1 |  | 0.1 | 0.4 | 0.1 | 0.2 |  |  | |  | |  | |  | |  | |  |
|  |  |  | fruit | 0.1 |  |  |  | 0.1 | 0.7 |  |  | |  | |  | |  | |  | |  |
|  | Apiaceae | *Daucus carota* | leave | 1.9 | 1.8 | 9 | 8.7 | 0.1 |  |  |  | |  | |  | | 0.5 | | 0.8 | | 2.0 |
|  | Urticaceae | *Urtica membranacea* | leave | 1.8 | 5.6 | 5.8 | 0.4 |  |  |  |  | |  | |  | | 5.3 | |  | | 4.5 |
|  |  |  | flower | 0.1 |  | 0.1 |  |  |  |  |  | |  | |  | |  | |  | | 1.5 |
|  |  | Eight undetermined species *^a^* | leave | 3.4 | 2.1 | 4.2 | 0.7 | 2.3 | 4.2 | 2.0 | 6.0 | | 3.3 | | 5.6 | | 5.3 | | 2.5 | | 2.0 |
|  |  | Two undetermined species *^a^* | fruit | + |  |  | 0.1 |  | 0.2 |  |  | |  | |  | |  | |  | |  |
|  |  | Mushrooms |  | 0.1 | 0.3 | 0.3 |  |  |  |  |  | |  | |  | |  | |  | |  |
| Animals |  | Ants |  | 0.6 | 1.8 | 1.4 | 2.1 | 0.7 | 0.2 | 0.4 | 0.5 | |  | |  | |  | |  | | 0.5 |
|  |  | Other insects |  | 0.4 | 0.3 |  | 0.6 | 0.6 | 2.6 |  | 0.5 | |  | |  | |  | |  | |  |
|  |  | Snails |  | 0.3 |  |  | 0.2 | 0 | 1.1 | 0.4 | 2.0 | |  | |  | |  | |  | |  |
| Food from humans |  | Fruits |  | 0.6 |  |  |  | 0.6 | 2.0 |  | 1.0 | | 0.8 | | 1.6 | | 0.5 | | 0.4 | |  |
|  |  | Peanuts |  | 0.1 |  |  |  | 0.5 | 0.4 |  |  | |  | |  | |  | |  | |  |
|  |  | Starch |  | 19.2 | 21.3 | 7.9 | 11.5 | 33.5 | 22.4 | 15.6 | 16.7 | | 15.5 | | 15.1 | | 22.4 | | 22.8 | | 25.2 |
| Water |  |  |  | 5.4 | 2.3 | 2.9 | 7.1 | 12.5 | 10.5 | 7.2 | 4.6 | | 3.4 | | 5.2 | | 4.3 | | 2.5 | | 2.5 |
| Number of plant species eaten |  |  |  | 77 | 26 | 55 | 51 | 37 | 25 | 21 | 24 | | 22 | | 18 | | 19 | | 27 | | 24 |
| Number of observations |  |  |  | 4864 | 341 | 736 | 901 | 936 | 446 | 249 | 197 | | 238 | | 193 | | 187 | | 241 | | 199 |
| Index of specific diversity |  |  |  | 13.8 | 10.8 | 18.0 | 20.0 | 7.5 | 10.1 | 5.6 | 11.2 | | 11.5 | | 9.5 | | 7.4 | | 7.6 | | 8.0 |

* : exotic species that monkeys found either in public or in private gardens. +: < 0.05% of the diet. Fruits provided by humans included grapes, apples, peers, melons, oranges, and chesnuts; starch included bread, pizzas and cakes. *^a^*: 1 to 10 undetermined species depending on the month. The phenology of each food item is indicated with a straight line under the cells of the table.
