# Supplementary material for: Is Diet Flexibility an Adaptive Life Trait for Relictual and Peri-Urban Populations of the Endangered Primate Macaca sylvanus?
Source: PLoS One. 2015 Feb 25;10(2):e0118596. doi: 10.1371/journal.pone.0118596 (PMC4340959; doi:10.1371/journal.pone.0118596)
Supplement: S2 Table — (DOCX) [file pone.0118596.s002.docx]

Table S2. Mean annual and monthly variations in the diet of non-urban Barbary macaque group ‘Cap Carbon’ at Gouraya National Park (Algeria), and phenology of food items.

|  |  |  |  | Years | 2007 |  |  |  |  |  |  |  |  | 2008 |  |  |
| --- | --- | --- | --- | --- | --- | --- | --- | --- | --- | --- | --- | --- | --- | --- | --- | --- |
|  | Family | Species | Plant part | Mean annual | A | M | J | J | A | S | O | N | D | J | F | M |
| Tree layer | Leguminosae | *Ceratonia siliqua* | bark | 0.3 |  |  | 0.6 | 0.4 |  |  |  |  |  | 2.1 |  |  |
|  |  |  | flower | 0.1 |  |  |  |  |  | 0.8 |  |  |  |  |  |  |
|  |  |  | fruit | 0.1 | 0.9 | 0.3 |  | 0.1 | 0.2 |  |  |  |  |  |  |  |
|  |  |  | seed | 1.2 |  | 11.2 | 3.4 |  |  |  |  |  |  |  |  |  |
|  | Oleaceae | *Olea europaea* | leave | 2.7 | 3.7 | 5.1 | 2.2 | 0.4 | 0.8 |  |  | 0.8 |  | 4.2 | 6 | 9.7 |
|  |  |  | flower | 1.7 | 0.3 |  |  |  |  |  |  |  |  |  | 7 | 12.9 |
|  |  |  | fruit | 8.4 |  |  |  |  |  | 5.5 | 17.9 | 22.3 | 28.6 | 26.1 | 0.4 |  |
|  |  |  | seed | 4.4 | 10.3 | 10.1 | 6.5 | 1.0 | 0.2 | 0.8 |  |  |  | 2.1 | 9.8 | 12.4 |
|  | Pinaceae | *Pinus halepensis* | bark | + |  |  |  |  |  |  |  | 0.4 |  |  |  |  |
|  |  |  | flower | 2.1 |  |  |  |  | 0.2 |  |  |  |  |  | 24.9 |  |
|  |  |  | seed | 17.4 |  |  | 17.2 | 13.9 | 20.8 | 46.6 | 45.5 | 30.1 | 22.7 | 7.7 | 3.2 | 0.5 |
| Shrub layer | Anacardiaceae | *Pistacia lentiscus* | leave | 0.1 |  |  | 0.3 |  | 0.4 |  |  | 0.4 |  |  |  |  |
|  |  |  | flower | 0.3 |  |  |  |  |  |  |  |  |  |  | 0.4 | 3.2 |
|  |  |  | fruit | 1.0 | 0.3 |  |  |  |  | 2.9 | 5.2 | 3.5 |  |  |  |  |
|  | Asparagaceae | *Asparagus albus* | buds | + | 0.3 |  |  |  |  |  |  |  |  |  |  |  |
|  | Cactaceae | *Opuntia* sp. *^b^* | fruit | 0.4 |  |  |  |  |  |  |  |  |  |  | 1.8 | 2.7 |
|  | Capparaceae | *Capparis spinosa* | fruit | + |  |  |  |  | 0.4 |  |  |  |  |  |  |  |
|  | Ericaceae | *Erica multiflora* | leave | + | 0.3 |  |  |  |  |  |  |  |  |  |  |  |
|  | Fabaceae | *Anagyris foetida* | flower | 0.2 |  |  |  |  |  |  |  |  | 1.8 |  |  |  |
|  |  | *Calicotome spinosa* | leave | 0.7 | 0.6 | 0.8 | 0.1 |  |  |  |  | 0.4 | 2.3 | 1.4 |  | 2.7 |
|  |  |  | flower | 0.1 | 1.6 |  |  |  |  |  |  |  |  |  |  |  |
|  | Fagaceae | *Quercus coccifera* | acorn | 7.0 | 7.8 | 5.1 | 3.3 |  |  |  | 1.9 | 8.6 | 10 | 16.2 | 22.1 | 9.1 |
|  |  |  | leave | 0.1 |  |  |  |  |  | 0.4 |  |  |  |  |  | 1.1 |
|  | Lamiaceae | *Teucrium flavum* | leave | + | 0.3 |  |  |  |  |  |  |  |  |  |  |  |
|  | Leguminoseae | *Acacia karroo* | leave | 4.4 | 9.6 | 12.6 | 9.7 | 7.6 | 3.6 | 3.4 | 0.7 | 0.8 | 1.8 |  | 0.7 | 2.7 |
|  |  |  | flower | 0.5 |  |  | 4.9 |  | 0.6 |  |  |  |  |  |  |  |
|  |  |  | seed | 0.1 | 1.2 |  |  |  |  |  |  |  |  |  |  |  |
|  | Malvaceae | *Lavatera olbia* | leave | + |  |  | 0.1 |  |  |  |  |  |  |  |  |  |
|  |  |  | flower | 0.1 |  | 0.6 |  |  |  |  |  |  |  |  |  |  |
|  |  |  | fruit | 0.1 |  | 1.1 |  |  |  |  |  |  |  |  |  |  |
|  | Moraceae | *Ficus carica* | leave | + |  |  |  |  |  |  |  |  |  |  |  | 0.5 |
|  |  |  | fruit | 0.1 |  |  |  |  | 0.8 |  |  |  |  |  |  |  |
|  | Oleaceae | *Jasminum fruticans* | leave | 0.1 |  | 0.3 | 0.3 |  |  |  |  |  |  |  |  |  |
|  |  | *Phillyrea angustifolia* | flower | 0.9 | 8.7 | 1.7 | 0.1 |  |  | 0.4 |  |  |  |  |  |  |
|  |  |  | fruit | 2.0 | 0.3 |  | 0.2 |  |  | 7.6 | 10.8 | 4.3 |  | 0.7 |  |  |
|  |  |  | seed | 1.7 | 8.4 | 3.1 | 1.2 | 0.1 | 0.2 | 0.4 |  |  |  | 0.7 | 4.6 | 1.1 |
|  |  | *Phillyrea latifolia* | flower | 0.7 |  |  |  |  |  |  |  |  | 8.6 |  |  |  |
|  | Rhamnaceae | *Rhamnus alaternus* | fruit | 0.1 |  |  | 0.6 |  |  |  |  |  |  |  |  |  |
|  |  | *Rhamnus lycioides* | fruit | 0.1 |  |  | 1 |  |  |  |  |  |  |  |  |  |
|  | Rosaceae | *Rosa sempervirens* | leave | 0.1 |  |  | 0.2 |  |  |  |  |  | 0.5 |  | 0.4 |  |
|  |  | *Rubus ulmifolius* | leave | + |  |  | 0.1 |  |  |  |  |  |  |  |  |  |
|  |  |  | fruit | + |  |  | 0.1 |  |  |  |  |  |  |  |  |  |
| Lianas | Caprifoliaceae | *Lonicera implexa* | stem | + | 0.3 |  |  |  |  |  |  |  |  |  |  |  |
|  | Ranunculaceae | *Clematis cirrhosa* | leave | + | 0.3 |  |  |  |  |  |  |  |  |  |  |  |
|  |  |  | flower | + |  |  |  |  |  |  |  |  | 0.5 |  |  |  |
|  |  | *Clematis flammula* | leave | 0.2 |  |  |  |  | 0.4 | 2.1 |  |  |  |  | 0.4 |  |
|  |  |  | flower | + |  |  | 0.4 | 0.1 |  |  |  |  |  |  |  |  |
|  | Rubiaceae | *Rubia peregrina* | leave | 0.1 | 0.6 |  |  |  |  |  |  |  | 0.5 |  |  |  |
|  | Smilacaceae | *Smilax aspera* | leave | 5.1 | 0.6 | 2.8 | 8.5 | 2.6 | 1.7 | 6.3 | 1.9 | 5.9 | 8.6 | 12.7 | 3.2 | 5.9 |
|  |  |  | fruit | 0.3 |  | 0.6 |  |  |  |  |  |  |  |  |  | 2.7 |
| Herbaceous layer | Araceae | *Arisarum vulgare* | stem | + |  |  |  |  |  |  |  |  |  |  | 0.4 |  |
|  | Asteraceae | *Calendula arvensis* | leave | 0.1 |  | 0.3 | 0.6 |  |  |  |  |  |  |  |  |  |
|  |  |  | flower | 0.3 | 0.3 | 2.8 | 0.4 |  |  |  |  |  |  |  |  |  |
|  |  | *Coleostephus myconis* | stem | 0.1 |  |  |  |  |  |  |  |  |  |  |  | 1.1 |
|  |  | *Galactites elegans* | fruit | + |  |  | 0.1 |  |  |  |  |  |  |  |  |  |
|  |  | *Helichrysum stoechas* | leave | 0.3 | 1.2 |  | 0.1 |  |  |  |  | 0.4 |  | 1.4 |  |  |
|  |  | *Pallenis maritima* | leave | + | 0.3 |  |  |  |  |  |  |  |  |  |  |  |
|  |  | *Phagnalon saxatile* | leave | 0.3 | 0.3 |  |  |  |  |  |  |  |  | 2.8 |  |  |
|  |  | *Sonchus oleraceus* | leave | 0.1 | 0.6 | 0.3 | 0.1 |  |  |  |  |  |  |  |  |  |
|  | Convolvulaceae | *Convolvulus althaeoides* | leave | 0.1 |  | 0.3 | 0.3 |  |  |  |  |  |  |  |  |  |
|  |  |  | flower | 0.1 |  | 0.6 |  |  |  |  |  |  |  |  |  |  |
|  |  | *Convolvulus sabatius* | leave | 0.1 | 0.3 | 0.3 |  |  |  |  |  |  |  |  |  |  |
|  | Crassulaceae | *Umbilicus rupestris* | stem | + | 0.3 |  |  |  |  |  |  |  |  |  |  |  |
|  | Cruciferae | *Sinapis arvensis* | leave | 0.4 | 0.9 | 0.6 |  |  |  |  |  |  |  |  |  | 2.7 |
|  | Cyperaceae | *Carex divisa* | leave | + |  | 0.3 | 0.1 |  |  |  |  |  |  |  |  |  |
|  | Geraniaceae | *Geranium robertianum* | leave | 0.1 |  | 0.6 |  |  |  |  |  |  |  |  |  |  |
|  |  |  | fruit | 0.1 |  | 0.6 |  |  |  |  |  |  |  |  |  |  |
|  |  | *Geranium rotundifolium* | leave | + |  |  | 0.1 |  |  |  |  |  |  |  |  |  |
|  |  |  | fruit | 0.1 |  | 0.8 | 0.2 |  |  |  |  |  |  |  |  |  |
|  | Lamiaceae | *Prasium majus* | leave | 0.1 |  |  |  |  |  |  | 0.4 |  | 0.5 |  |  |  |
|  | Leguminosae | *Tripodion tetraphyllum* | leave | 0.1 |  | 0.8 | 0.1 |  |  |  |  |  |  |  |  |  |
|  | Malvaceae | *Malva sylvestris* | leave | 0.3 | 1.2 | 1.4 | 0.1 |  |  |  |  |  | 0.5 |  | 0.4 |  |
|  |  |  | fruit | 0.2 |  | 2.8 |  |  |  |  |  |  |  |  |  |  |
|  |  |  | seed | 0.1 |  |  | 0.7 |  |  |  |  |  |  |  |  |  |
|  | Oxalidaceae | *Oxalis pes-caprae* | leave | 0.2 |  | 0.3 |  |  |  |  |  | 0.4 |  | 0.7 | 0.7 |  |
|  |  |  | flower | + | 0.3 |  |  |  |  |  |  |  |  |  |  |  |
|  |  |  | root | 0.2 | 0.6 |  |  | 0.2 | 0.4 |  |  |  |  |  | 1.1 |  |
|  | Plantaginaceae | *Plantago lagopus* | leave | 0.1 | 0.6 | 0.3 |  |  |  |  |  |  |  |  |  |  |
|  |  | *Plantago major* | leave | 0.1 |  |  |  |  |  |  |  | 0.8 |  |  |  |  |
|  | Poaceae | *Ampelodesmos mauritanicus* | leave | 0.5 |  |  |  |  |  |  |  |  | 0.5 | 0.7 | 0.4 | 3.8 |
|  |  | *Brachypodium sylvaticum* | leave | 2.3 | 8.7 | 1.4 | 1 |  |  |  |  | 0.8 | 0.5 | 3.5 | 1.4 | 10.2 |
|  |  | *Catapodium rigidum* | leave | 0.5 | 3.4 |  | 0.2 |  |  |  |  | 0.4 |  |  |  | 1.6 |
|  |  | *Cynodon dactylon* | leave | + |  |  | 0.1 |  |  |  |  |  |  |  |  |  |
|  |  | *Hordeum murinum* | leave | + |  | 0.3 |  |  |  |  |  |  |  |  |  |  |
|  |  | *Hyparrhenia hirta* | leave | 0.1 |  | 0.6 | 0.3 |  |  |  |  |  |  |  |  |  |
|  |  |  | flower | + |  |  | 0.1 |  |  |  |  |  |  |  |  |  |
|  |  | *Cenchrus setaceus* | leave | 4.6 | 7.1 | 6.7 | 4.5 | 1.3 | 3.6 | 5.8 | 6.7 | 6.3 | 4.5 | 5.6 | 1.1 | 2.2 |
|  |  | *Piptatherum miliaceum* | leave | 0.1 |  |  | 0.3 |  |  |  |  |  |  |  | 0.4 |  |
|  | Primulaceae | *Lysimachia monelli* | fruit | 0.2 |  | 2.8 |  |  |  |  |  |  |  |  |  |  |
|  | Resedaceae | *Reseda alba* | leave | 0.1 | 0.6 |  |  |  |  |  |  |  |  |  |  |  |
|  | Ruscaceae | *Ruscus hypophyllum* | fruit | 0.1 |  |  |  |  |  |  |  |  |  | 0.7 |  |  |
|  | Solanaceae | *Solanum nigrum* | leave | + |  |  |  |  |  |  |  |  |  |  | 0.4 |  |
|  | Apiaceae | *Daucus carota* | leave | 0.3 | 2.2 | 0.3 |  |  |  |  |  |  |  |  | 0.4 | 0.5 |
|  | Urticaceae | *Urtica membranacea* | leave | 0.1 | 0.6 |  | 0.1 |  |  |  |  |  |  |  | 0.4 | 0.5 |
|  | Valerianaceae | *Centranthus ruber* | flower | 0.1 |  | 0.6 | 0.4 |  |  |  |  |  |  |  |  |  |
|  |  | Three undetermined species *^a^* | leave | 1.0 | 2.2 | 1.4 |  |  |  |  |  | 0.8 | 1.4 | 2.1 | 2.1 | 2.2 |
|  |  | Mosses |  | + | 0.3 |  |  |  |  |  |  |  |  |  |  |  |
|  |  | Mushrooms |  | 1.0 | 0.3 |  | 0.3 |  |  |  | 2.6 | 8.6 |  |  |  |  |
| Animals |  | Ants |  | 0.3 | 1.2 | 0.8 | 1.7 |  |  |  |  |  |  |  |  |  |
|  |  | Other insects |  | 0.1 |  | 0.6 | 1.1 |  |  |  |  |  |  |  |  |  |
|  |  | Birds |  | + |  |  | 0.1 |  |  |  |  |  |  |  |  |  |
| Food from humans |  | Fruits |  | 1.4 | 0.6 |  | 0.5 | 6.2 | 7.6 | 1.7 |  |  |  |  | 0.4 |  |
|  |  | Peanuts |  | 2.1 | 2.5 | 2.8 | 1.4 | 6.7 | 8.6 |  |  |  |  | 1.4 | 0.4 | 1.1 |
|  |  | Starch |  | 10.9 | 3.1 | 7.9 | 16.4 | 40.7 | 29.6 | 6.7 | 2.3 | 3.6 | 2.8 | 6.3 | 5 | 5.9 |
| Water |  |  |  | 6.2 | 3.4 | 5.6 | 7 | 18.5 | 19.7 | 8.4 | 4.1 | 0.8 | 3.6 | 0.7 | 1.1 | 1.1 |
| Number of plant species eaten |  |  |  | 65 | 32 | 29 | 33 | 9 | 12 | 10 | 9 | 15 | 16 | 16 | 22 | 19 |
| Number of observations |  |  |  | 4441 | 322 | 356 | 891 | 801 | 476 | 238 | 268 | 256 | 220 | 142 | 285 | 186 |
| Index of specific diversity |  |  |  | 10.4 | 11.6 | 13.4 | 10.1 | 5.0 | 6.7 | 4.1 | 3.8 | 5.8 | 6.1 | 6.1 | 5.3 | 6.2 |

+: < 0.05% of the diet. Fruits provided by humans included grapes, apples, peers, watermelons, melons, and bananas; peanuts included also pistachios; starch included bread, pizzas and cakes. *^a^*: 0 to 3 undetermined species depending on the month. The phenology of each food item is indicated with a straight line under the cells of the table.
