# Supplementary material for: Is Diet Flexibility an Adaptive Life Trait for Relictual and Peri-Urban Populations of the Endangered Primate Macaca sylvanus?
Source: PLoS One. 2015 Feb 25;10(2):e0118596. doi: 10.1371/journal.pone.0118596 (PMC4340959; doi:10.1371/journal.pone.0118596)
Supplement: S1 Appendix — (DOCX) [file pone.0118596.s004.docx]

Appendix S4. Negative effects of urban colonization by Barbary macaques on human property and on monkeys.

This appendix provides examples of nuisance to humans when monkeys colonize a city and some of the negative effects for monkeys.

Monkeys are bin-raiders because of their high manual handling capacity and are often reported foraging in garbage and turning over bins in streets and parks of Bejaia suburban zones (Fig. S1). They use roofs as refuges, cause damage to property, and this causes people to complain (Fig. S2).

Figure S1: Monkeys foraging in a bin.


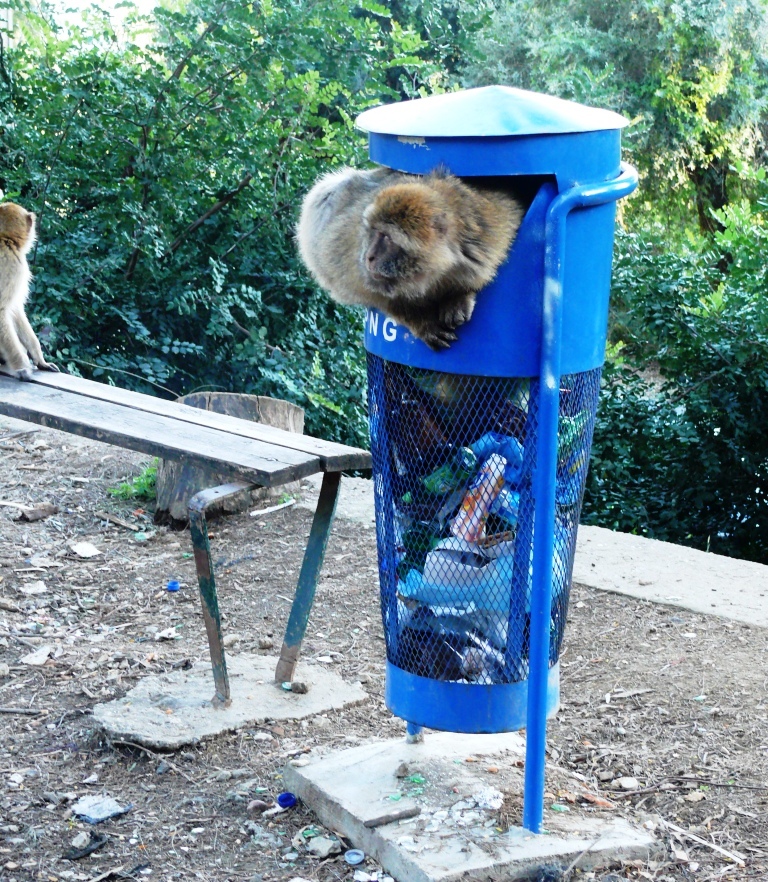
©Nelly Ménard, CNRS

Figure S2: Monkeys walking on roofs.


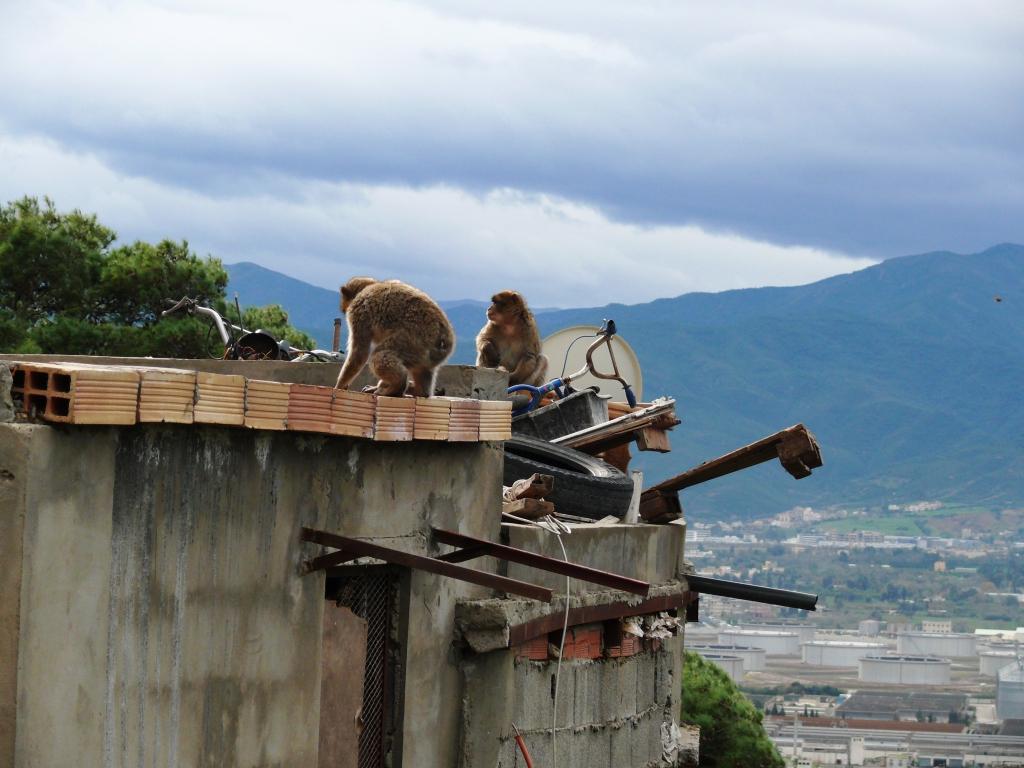
©Nelly Ménard, CNRS

Local residents respond to monkeys’ damage and raiding behaviour by aggression (*e.g*. slingshot-shooting), and this induces many injuries such as gouged eyes (Fig. S3) or fingers partly snatched by traps.

Figure S3: Monkeys with gouged eyes.


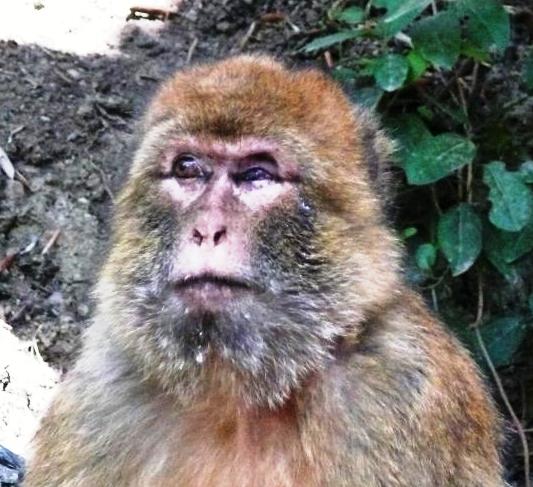
©Nelly Ménard, CNRS


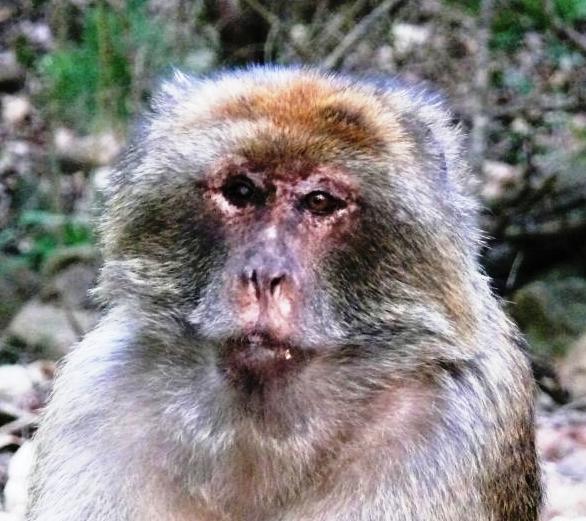
©Nelly Ménard, CNRS

Some monkeys get overweight on anthropogenic food (Fig. S4).

Figure S4. One adult male of the Oliv group showing clear signs of obesity.


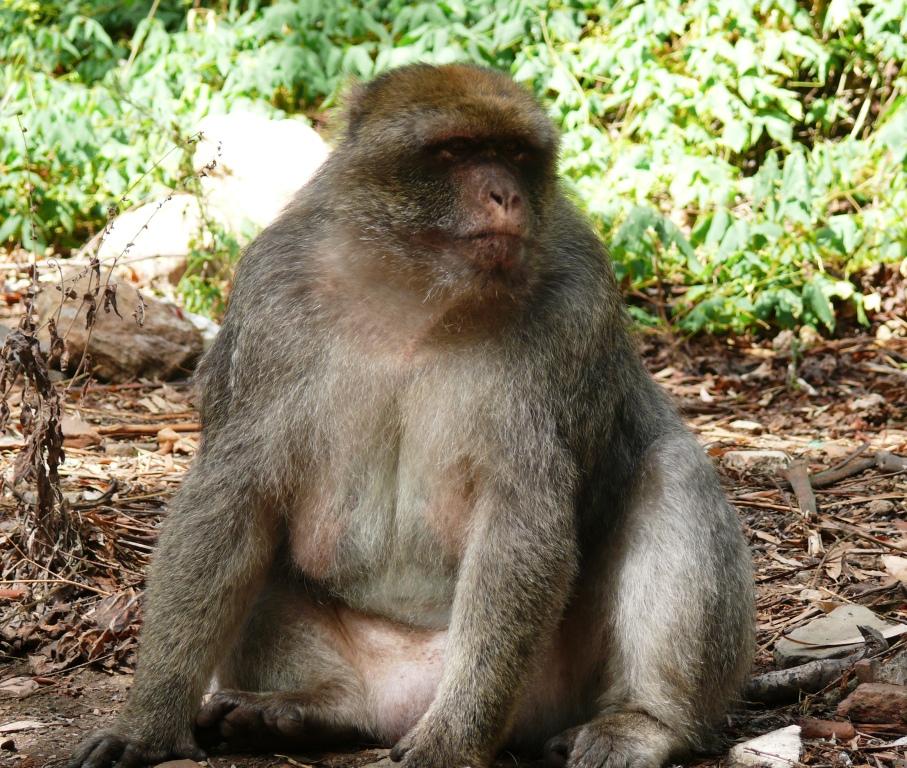
©Nelly Ménard, CNRS

Barbary macaques often use electric wires and some are killed by electrocution (Fig. S5).

Figure S5: “Petit gris”, a 5 years old male, received an electric shock while walking on an electric wire. He was violently thrown down to the ground from more than 20m high. He stayed motionless more than one hour and then only started moving very slowly. The next day, he was with his group, exhibiting signs of severe burning.


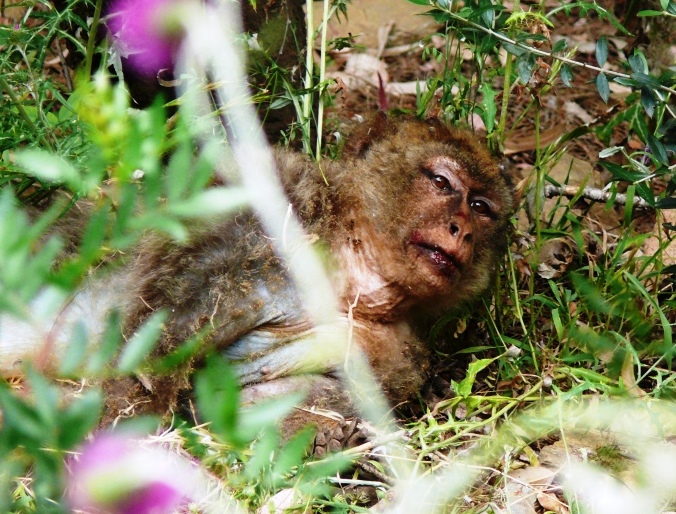
©Nelly Ménard, CNRS


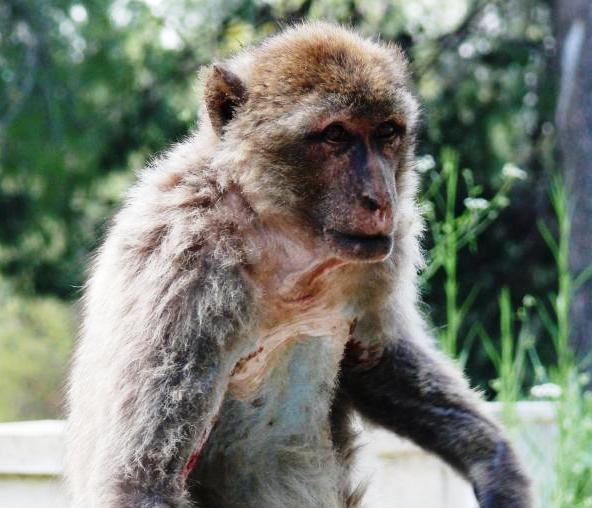
©Nelly Ménard, CNRS

Wild populations in which fear of humans is lower can turn into special targets for poachers. Infants can be caught to be used and sold as pets. Zino’s story illustrates this possibility. Zino was 3 years old when he was observed in one of the Gouraya groups. He had a nylon rope around his body that had probably been there for a long time because the rope had gone deep into his flesh as he grew taller and bigger, provoking infected wounds (Fig. S6). He had probably been caught as an infant as usually occurs (that was two years before) and had possibly escaped or had been released. The rope limited his growth and would probably have caused him to die in agony. Thanks to the initiative of two veterinarians of Chrea Park in Algeria who collaborated with Gouraya Park, Zino was successfully caught and operated on. His health was monitored for a month at Chréa before he was released back into his own group at Gouraya (Fig. S7).

Figure S6: Zino with a nylon rope provoking infectious injuries to his body.
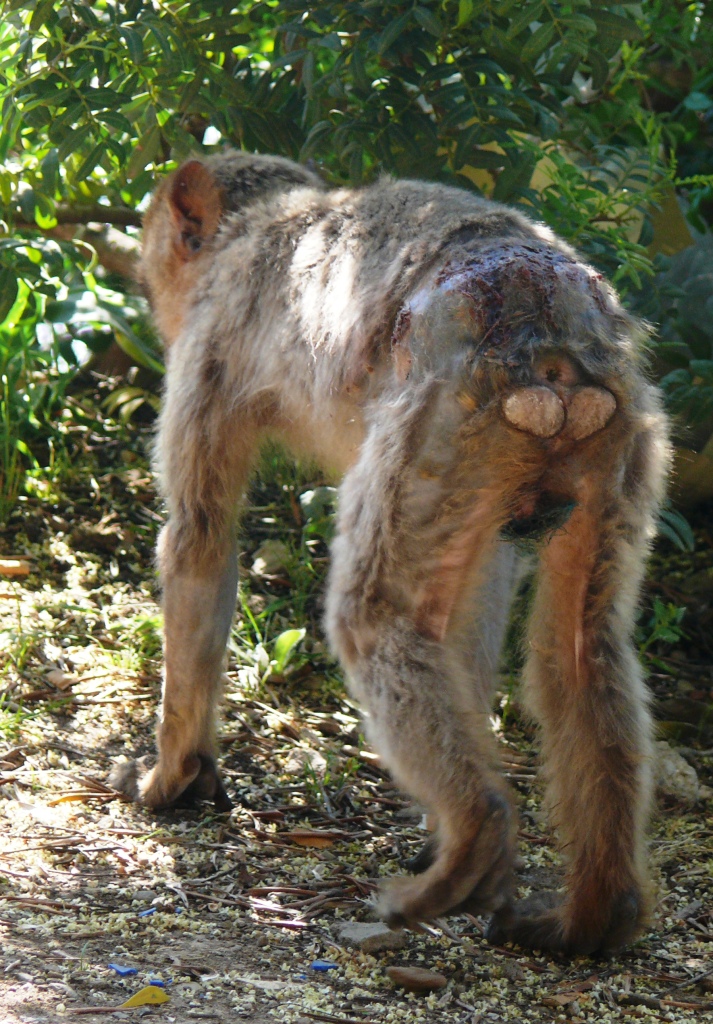


©Nelly Ménard, CNRS

Figure S7: Zino five months after he was released into his group.
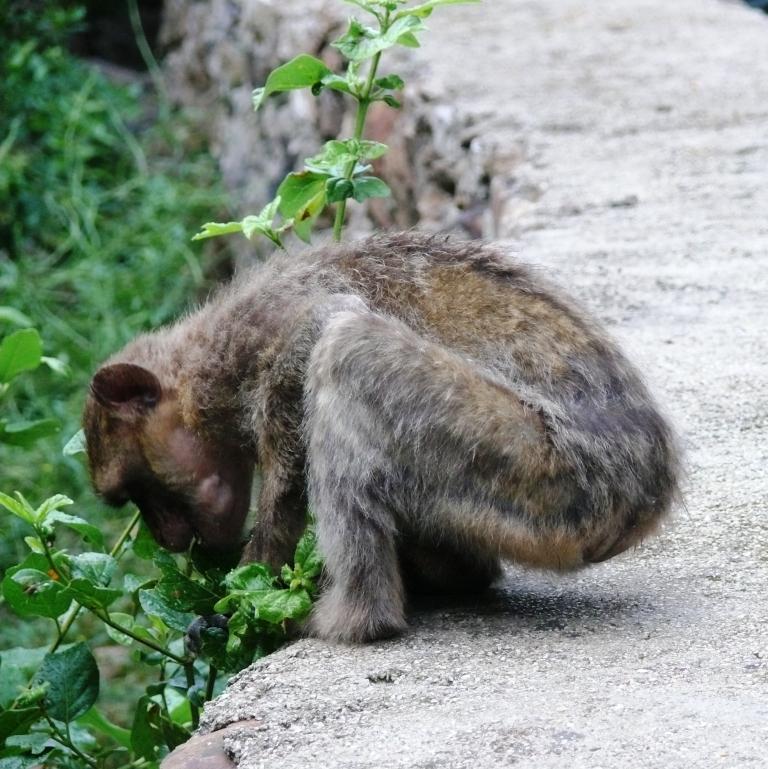
©Nelly Ménard, CNRS
